# Defining characteristics predicting mental health disorders among undergraduate students in the post-COVID-19 era: a multicenter cross-sectional study*

**DOI:** 10.1590/1518-8345.7741.4671

**Published:** 2025-10-27

**Authors:** Caroline de Castro Moura, Luana Vieira Toledo, Gabriela Tavares Boscarol, Rafael Lopes Chaves, Denismar Alves Nogueira, Carine Alves da Costa Marinho, Bruna de Oliveira Alves, Bárbara Guimarães Lourenço, Érika de Cássia Lopes Chaves, Tânia Couto Machado Chianca

**Affiliations:** 1 Universidade Federal de Viçosa, Departamento de Medicina e Enfermagem, Viçosa, MG, Brazil.; 2 Universidade Federal de Minas Gerais, Escola de Enfermagem, Belo Horizonte, MG, Brazil.; 3 Scholarship holder at the Coordenação de Aperfeiçoamento de Pessoal de Nível Superior (CAPES), Brazil.; 4 Universidade Professor Edson Antônio Velano, Curso de Psicologia, Alfenas, MG, Brazil.; 5 Scholarship holder at the Fundação de Amparo à Pesquisa do Estado de Minas Gerais (FAPEMIG), Brazil.; 6 Universidade Federal de Alfenas, Departamento de Estatística, Alfenas, MG, Brazil.; 7 Universidade Federal de Alfenas, Escola de Enfermagem, Alfenas, MG, Brazil.

**Keywords:** Signs and Symptoms, Mental Health, Universities, Students, COVID-19, Nursing

## Abstract

to evaluate defining characteristics of nursing diagnoses that are predictors of mental health disorders among undergraduate students in the post-COVID-19 era.

cross-sectional, multicenter study conducted with undergraduate students from four public universities in the state of Minas Gerais. Data were collected through a structured, online questionnaire with sociodemographic and clinical variables. The presence of defining characteristics included in the Nursing Diagnoses of Anxiety, Change Stress Syndrome; Post-Trauma Syndrome; Stress Overload and Chronic Sadness, present in the NANDA-I taxonomy, was also investigated through self-report. The Depression, Anxiety and Stress Scale and the Impact of Events Scale-Revised were also used. Multivariate logistic regression analysis was performed to determine the defining characteristics that were configured as predictors of mental health disorders. Adjusted odds ratios were estimated, along with 95% confidence intervals, at 5% significance.

2,349 undergraduate students participated in the study. The presence of 24 physical, behavioral, cognitive, and emotional defining characteristics of the nursing diagnoses in question was investigated. Of these, 13 emerged as predictors for anxiety symptoms; 15 for stress symptoms; 11 for depression symptoms; and 15 for post-traumatic stress disorder.

physical, behavioral, cognitive, and emotional defining characteristics of NANDA-I nursing diagnoses were evidenced as predictors for the development of mental health disorders in university students in the post-COVID-19 era.

## Highlights

**(1)** Mental health disorders are prevalent among college students in the post-COVID-19 era. **(2)** Nursing diagnoses related to mental health are prevalent among students. **(3)** Defining characteristics have emerged as predictors of mental health disorders. **(4)** Interventions to promote mental health in this population are needed.

## Introduction

According to the World Health Organization, mental health is defined as “a state of well-being in which the individual is aware of his or her own abilities, can cope with everyday stresses, works productively, and is able to contribute to his or her community”^(1)^. Disorders related to it represent a major public health problem worldwide and arise when a person is unable to adapt to the changes that occur in his or her life, causing great suffering^(1)^.

Across the world, one in eight people live with mental health disorders^(1)^. The results of the Global Burden of Diseases showed that between 1990 and 2019, the global number of disability-adjusted life years due to mental disorders increased from 80.8 million to 125.3 million, with a great burden on people around the world^(2)^.

Undergraduate students represent a population vulnerable to the development of mental health disorders^(3)^, and the COVID-19 pandemic has further aggravated them^(4-5)^, as among its numerous negative impacts, it has triggered a global crisis in people’s mental health^(1)^. In fact, anxiety, stress, depression, and post-traumatic stress disorder (PTSD) have remained prevalent in the post-COVID-19 era^(6-7)^.

In the context of nursing, the NANDA-I taxonomy^(8)^ presents some diagnoses related to mental health that may be present in the university population, and have probably been intensified due to the pandemic context. Among them, the following stand out: Anxiety (00146); Stress Overload (00177); Post-Trauma Syndrome (00141); Change Stress Syndrome (00114), and Chronic Sadness (00137). Paying attention to the defining characteristics and related factors that can trigger these problems, in order to implement effective measures to solve and prevent them, is essential in this context of mental health crisis.

Globally, studies have already evaluated mental health disorders in university students in the post-COVID-19 era^(6-7,9-12)^. For example, a study conducted in China^(6)^ identified that factors associated with social, family and economic changes imposed by the pandemic increased the risk of psychological symptoms in university students. Another study^(7)^, conducted in Chile, identified, in addition to high prevalences of depression, anxiety and stress, that female gender, belonging to sexual minorities and use of psychotropic drugs seem to have an impact on susceptibility to mental health problems. And a study conducted in Thailand^(12)^ found that medical conditions, poor relationships with family, friends or other people, having problems while studying at university and self-perceived impact of COVID-19 on the student’s life were all associated with mental health problems.

It is noteworthy that most studies have mainly involved tracking psychological status and its association with demographic, clinical, and academic factors^(6-7,9-12)^. However, research in Brazil is scarce and, to date, no systematic or scoping review studies have been found, at national or international level, or that have evaluated the presence of defining characteristics of nursing diagnoses in undergraduate students in the post-COVID-19 era, and estimated them as predictors of anxiety, stress, depression, and PTSD. Assessing these characteristics in a population vulnerable to the development of mental health disorders and that has experienced a major health crisis can be an important step in the development of institutional care strategies for this population. In this sense, the present study aimed to evaluate defining characteristics of nursing diagnoses that are configured as predictors of mental health disorders among undergraduate students in the post-COVID-19 era.

## Method

### Study design

A cross-sectional, multicenter study was conducted with undergraduate university students and reported in accordance with the recommendations of Strengthening the Reporting of Observational Studies in Epidemiology^(13)^.

### Setting and period

The study was carried out at four public universities in the state of Minas Gerais, Brazil, between January 2023 and April 2024. University 1 has 79 undergraduate courses distributed across five campuses in the Metropolitan and Northern regions of the state of Minas Gerais; University 2 has 56 undergraduate courses, across three campuses in the Metropolitan region; University 3 has 67 undergraduate courses across three campuses in *Zona da Mata*, the Metropolitan region and *Alto Paranaíba*/*Triângulo Mineiro*; finally, University 4 is made up of 38 undergraduate courses across four campuses, located in the South and Southwest of the state. Population and sample definition

The population consisted of 67,110 undergraduate university students, 33,900 at university 1, 13,110 at university 2, 14,000 at university 3, and 6,100 at university 4. To calculate the sample, a conservative prevalence estimate of 50% was used, since the prevalence of mental health disorders in the post-COVID-19 era in Brazil was unknown at the time of planning the study. The formula n = (z2.p(1-p)/e2)/1+(z2.p(1-p)/e2N) was used, where: z = z-score (for 95% confidence level, z = 1.96); p = prevalence of cases with the characteristic studied; e = margin of error; N = population size. Considering the population of 67,110 students and a margin of error of 2%, the minimum sample size estimated for the present study was 2,319 students.

### Selection criteria

Students aged 18 or over, who were regularly enrolled in any period of undergraduate courses and who had time available to respond to the data collection instruments were recruited. Those who did not have access to the internet at the time of data collection and who did not complete the entire survey were excluded from the study.

### Study variables

The presence or absence of symptoms of anxiety, stress, depression and PTSD were considered dependent variables. The defining characteristics of the nursing diagnoses studied were considered explanatory or predictive. Sociodemographic and clinical characteristics were considered covariates.

### Instruments used to collect information

The following sociodemographic and clinical characteristics were collected through a structured online questionnaire: biological sex; skin color; marital status; housing; family income (in Brazilian minimum wages - at the time of data collection: R$1,320.00 – Brazilian currency - *reais*); work activity; student assistance; student grant (for teaching, research or extension); clinical diagnosis of mental health disorders; use of psychotropic drugs; follow-up with a psychiatrist and psychologist. In addition, the presence of defining characteristics of the following nursing diagnoses was investigated through self-report, according to the NANDA-I taxonomy^(8)^:

1) Anxiety: psychomotor agitation; easy crying; insecurity; alteration in the sleep-wake cycle; decreased productivity; tightness in the chest; dry mouth; diarrhea; muscle tension; heart palpitations; nausea; altered breathing pattern; facial flushing; increased sweating; altered attention; forgetfulness; worry; fear.
2) Change Stress syndrome: low self-esteem; anger; fear; worry; frustration; alteration in the wake-wake cycle; loneliness.
3) Stress overload: anger; increased impatience; muscle tension.
4) Post-trauma syndrome: altered attention; headache; anger; fear; heart palpitations.
5) Chronic sadness: sadness.

These defining characteristics were selected because they were observed more frequently in undergraduate students, according to the clinical practice of six researchers on the team, who are nurses and university professors. In addition, some of these defining characteristics are also present in the Depression, Anxiety, and Stress Scale (DASS-21)^(14)^ and the Impact of Events Scale-Revised (IES-R)^(15)^.

The DASS-21^(14)^ was used to measure symptoms of anxiety, stress, and depression. The scale has 21 questions, in the form of self-report, divided into three subscales that assess symptoms perceived in the last week, through seven questions, with four answer options (0 = did not apply at all to 3 = applied very much or most of the time). The sum of each subscale, multiplied by two, provides the total score for each construct^(14)^. The scale was translated and validated for the Brazilian version and has adequate psychometric properties^(14)^. In the present sample, the overall Cronbach’s alpha was 0.944 (for the anxiety, stress and depression subscales it was 0.867, 0.886 and 0.901, respectively)^(16)^.

The IES-R^(15)^ was used to assess PTSD. The IES-R is self-administered and has 22 items subdivided into three symptom subscales: avoidance, intrusion and hyperarousal, which consider the last seven days. Each item comprises five response options (0- not at all to 4- extremely). The overall result is the sum of the means of each subscale. A value greater than 5.6 indicates the presence of PTSD^(15)^. The instrument was validated and adapted for the Brazilian version^(15)^, and in the present sample, Cronbach’s alpha was 0.961^(16)^.

### Data collection

The study was conducted online, via the Google Forms platform, over three semesters, in order to reach the number of participants estimated by the sample size calculation. The study was publicized through the universities’ official communication channels, institutional emails and through the distribution of pamphlets. We provided all potential participants with detailed information about the research objectives so that they could make informed decisions about whether or not to participate. Those who agreed signed the informed consent form in digital format. They then responded, through self-reporting, to the data collection instruments. The data were stored in a password-protected electronic format.

### Data processing and analysis

A database was generated using the responses obtained through Google Forms in Microsoft Office Excel, version 2021. The data were imported into IBM SPSS Statistics for Windows, version 26 (IBM Corp., Armonk, NY, USA) for analysis. Relative and absolute frequencies, mean, and standard deviation summarized the variables studied. Bivariate analysis using Pearson’s chi-square test was used to assess the association between the defining characteristics of the nursing diagnoses studied and the symptoms of anxiety, stress, depression, and PTSD assessed on the scales used.

For the multiple regression analysis, the multicollinearities of the variables were also assessed. The VIF test for collinearity showed values close to 1, showing no multicollinearity problem. The presence or absence of symptoms of anxiety, stress, depression, and PTSD were considered dependent variables. The explanatory variables (defining characteristics of the nursing diagnoses studied) were inserted using the Backward method into the multiple logistic regression model. The adjusted odds ratios (AOR) were estimated, along with their 95% confidence intervals (95% CI), which were used to determine those defining characteristics that could be configured as predictors of symptoms of anxiety, stress, depression and PTSD. The association of variables was considered statistically significant for p<0.05.

The sociodemographic and clinical variables were placed in the group of variables of the multiple logistic regression model; however, as they were not statistically significant (p>0.05), it was decided to maintain only the defining characteristics of the nursing diagnoses in question.

## Ethical aspects

Ethical standards for research involving human beings were strictly followed. The study was approved by the Research Ethics Committee under opinion number 5.700.107/CAAE: 63318222.0.0000.5153. All students who agreed to participate in the study signed the Free and Informed Consent Form.

## Results

A total of 2,409 undergraduate students were evaluated, of whom 97.5% (2,349) completed the study. 2.5% (60) were excluded from the analysis because they were graduate students (48); refused to participate in the study (7); did not respond to the survey completely (3); or were under 18 years old (2).

The participants’ ages ranged from 18 to 62 years, with a mean of 23.4 years (standard deviation: 5.4). 48.6% (1,141) were enrolled in courses in the area of biological and health sciences; 24.2% (569) in human sciences, arts and letters; 21.3% (501) in exact and technological sciences; and 5.9% (138) in agricultural sciences. The description of the sociodemographic and clinical characteristics is shown in Table 1.

**Table 1-:** Sociodemographic and clinical characteristics of undergraduate university students (n = 2,349). Viçosa, MG, Brazil, 2024

**Variables**	f*	**(%** ^†^ **)**
**Gender**		
Male	572	24.4
Female	1.777	75.6
**Skin color**		
White	1.353	57.6
Yellow	27	1.1
Brown	676	28.8
Black	287	12.2
Indigenous	6	0.3
**Marital status**		
Single	2.203	93.8
Married/stable union	132	5.6
Widower	1	0.1
Divorced	13	0.6
**Living**		
Alone	410	17.5
With Family	942	40.1
Pension	58	2.5
Shared house	794	33.8
student accommodation	145	6.2
**Family income in minimum wages** ^‡^		
Up to 3	1.380	58.7
4 or more	969	41.3
**Current work activity**	606	25.8
**Student assistance at the moment**	621	26.4
**Student scholarship at the moment**	476	20.3
**Clinical diagnosis of mental health disorders**	1.337	56.9
**Use of psychotropic drugs**	956	40.7
**Follow-up with a psychiatrist**		
No	1.638	69.7
I already did it before the pandemic	302	12.9
I started doing it during/after the pandemic	409	17.4
**Follow-up with a psychologist**		
No	1.254	53.4
I already did it before the pandemic	461	19.6
I started doing it during/after the pandemic	634	27.0

*f = Absolute frequency; ^†^% = Relative frequency; ^‡^Minimum wage value at the time of data collection: R$1,320.00 *reais* (Brazilian currency), in 2023

Among the 2,349 students who participated in the study, 76.8% (1,804) had symptoms of anxiety; 77.3% (1,816) symptoms of stress; 77.2% (1,814) symptoms of depression; and 42.2% (994) PTSD, which were measured by the scales used.

The bivariate analysis demonstrated that all the defining characteristics of the nursing diagnoses selected for measurement and reported by the students were statistically significantly associated with symptoms of anxiety, stress, depression, and PTSD in the post-COVID-19 era (Table 2).

Table 3 presents the defining characteristics that were configured as predictors of mental health disorders among undergraduate university students based on multivariate logistic regression models.

**Table 2-:** Association between defining characteristics reported by undergraduate university students and the presence of symptoms of anxiety, stress, depression and post-traumatic stress disorder, in the post-COVID-19 era (n = 2,349). Viçosa, MG, Brazil, 2024

		**Anxiety symptoms**	**p-value**	**Stress symptoms**	**p-value**	**Depression symptoms**	**p-value**	Post-traumatic stress disorder f*(% ^†^ **)**	**p-value**
		f* (% ^†^ **)**		f* (% ^†^ **)**		f* (% ^†^ **)**			
**Physical defining characteristics**									
Change in the sleep-wake cycle ^‡§^	No	329 (18.2)	**<0.001**	326 (18.0)	**<0.001**	335 (18.5)	**<0.001**	130 (13.1)	**<0.001**
	Yes	1475 (81.8)		1490 (82.0)		1479 (81.5)		864 (86.9)	
Increased sweating ^‡^	No	900 (49.9)	**<0.001**	917 (50.5)	**<0.001**	940 (51.8)	**<0.001**	433 (43.6)	**<0.001**
	Yes	904 (50.1)		899 (49.5)		874 (48.2)		561 (56.4)	
Dry mouth ^‡^	No	825 (45.7)	**<0.001**	871 (48.0)	**<0.001**	882 (48.6)	**<0.001**	391 (39.3)	**<0.001**
	Yes	979 (54.3)		945 (52.0)		932 (51.4)		603 (60.7)	
Altered breathing pattern ^‡^	No	636 (35.3)	**<0.001**	657 (36.2)	**<0.001**	701 (38.6)	**<0.001**	284 (28.6)	**<0.001**
	Yes	1168 (64.7)		1159 (63.8)		1113 (61.4)		710 (71.4)	
Heart palpitations ^‡||^	No	644 (35.7)	**<0.001**	694 (38.2)	**<0.001**	720 (39.7)	**<0.001**	308 (31.0)	**<0.001**
	Yes	1160 (64.3)		1122 (61.8)		1094 (60.3)		686 (69.0)	
Muscle tension ^‡¶^	No	373 (20.8)	**<0.001**	362 (19.9)	**<0.001**	408 (22.5)	**<0.001**	168 (16.9)	**<0.001**
	Yes	1431 (79.3)		1454 (80.1)		1406 (77.5)		826 (83.1)	
Headache ^||^	No	641 (35.5)	**<0.001**	646 (35.6)	**<0.001**	6607 (36.8)	**<0.001**	296 (29.8)	**<0.001**
	Yes	1163 (64.5)		1170 (64.4)		1147 (63.2)		698 (70.2)	
Nausea ^‡||^	No	1080 (59.9)	**<0.001**	1101 (60.6)	**<0.001**	1117 (61.6)	**<0.001**	545 (54.8)	**<0.001**
	Yes	724 (40.1)		715 (39.4)		697 (38.4)		449 (45.2)	
Tightness in the chest ^‡^	No	591 (32.8)	**<0.001**	625 (34.4)	**<0.001**	646 (35.6)	**<0.001**	264 (26.6)	**<0.001**
	Yes	1213 (67.2)		1191 (65.6)		1168 (64.4)		730 (73.4)	
Diarrhea ^‡||^	No	1235 (68.5)	**<0.001**	1245 (68.6)	**<0.001**	1260 (69.5)	**<0.001**	640 (64.4)	**<0.001**
	Yes	569 (31.5)		571 (31.4)		554 (30.5)		354 (994)	
Facial flushing ^‡^	No	1427 (79.1)	**<0.001**	1441 (79.4)	**<0.001**	1453 (80.1)	**<0.001**	756 (76.1)	**<0.001**
	Yes	373 (20.7)		371 (20.4)		357 (19.7)		238 (23.9)	
**Behavioral defining characteristics**									
Psychomotor agitation ^‡^	No	150 (8.3)	**<0.001**	131 (7.2)	**<0.001**	180 (9.9)	**<0.001**	61 (6.1)	**<0.001**
	Yes	1654 (91.7)		1685 (92.8)		1634 (90.1)		933 (93.9)	
Easy cry ^‡^	No	490 (27.2)	**<0.001**	483 (26.6)	**<0.001**	521 (28.7)	**<0.001**	214 (21.5)	**<0.001**
	Yes	1314 (72.8)		1333 (73.4)		1293 (71.3)		780 (78.5)	
Increased impatience ^¶^	No	219 (12.1)	**<0.001**	169 (9.3)	**<0.001**	214 (11.8)	**<0.001**	80 (8.0)	**<0.001**
	Yes	1585 (87.9)		1647 (90.7)		1600 (88.2)		914 (92.0)	
Decreased productivity ^‡^	No	287 (15.9)	**<0.001**	292 (16.1)	**<0.001**	258 (14.2)	**<0.001**	123 (12.4)	**<0.001**
	Yes	1517 (84.1)		1524 (83.9)		1556 (85.8)		871 (87.6)	
Low self-esteem ^§^	No	266 (14.9)	**<0.001**	273 (15.1)	**<0.001**	246 (13.7)	**<0.001**	96 (9.7)	**<0.001**
	Yes	1525 (85.1)		1531 (84.9)		1556 (86.3)		898 (90.3)	
**Cognitive defining characteristics**									
Altered attention ^‡||^	No	212 (11.8)	**<0.001**	201 (11.1)	**<0.001**	209 (11.5)	**<0.001**	87 (8.8)	**<0.001**
	Yes	1592 (88.2)		1615 (88.9)		1605 (88.5)		907 (91.2)	
Frequent forgetfulness ^‡^	No	291 (16.1)	**<0.001**	280 (15.4)	**<0.001**	296 (16.3)	**<0.001**	117 (11.8)	**<0.001**
	Yes	1513 (83.9)		1536 (84.6)		1518 (83.7)		877 (88.2)	
**Emotional defining characteristics**									
Excessive worry ^‡§^	No	110 (6.1)	**<0.001**	96 (5.3)	**<0.001**	126 (6.9)	**<0.001**	24 (2.4)	**<0.001**
	Yes	1694 (93.9)		1720 (94.7)		1688 (93.1)		970 (97.6)	
Frustration ^§^	No	195 (10.8)	**<0.001**	190 (10.5)	**<0.001**	154 (8.5)	**<0.001**	68 (6.8)	**<0.001**
	Yes	1609 (89.2)		1626 (89.5)		1660 (91.5)		926 (93.2)	
Loneliness ^§^	No	437 (24.2)	**<0.001**	421 (23.2)	**<0.001**	401 (22.1)	**<0.001**	178 (17.9)	**<0.001**
	Yes	1367 (75.8)		1395 (76.8)		1413 (77.9)		816 (82.1)	
Fear ^‡§||^	No	305 (16.9)	**<0.001**	326 (18.0)	**<0.001**	325 (17.9)	**<0.001**	114 (11.5)	**<0.001**
	Yes	1499 (83.1)		1490 (82.0)		1489 (82.1)		880 (88.5)	
Anger ^§||¶^	No	557 (30.9)	**<0.001**	521 (28.7)	**<0.001**	538 (29.7)	**<0.001**	225 (22.6)	**<0.001**
	Yes	1247 (69.1)		1295 (71.3)		1276 (70.3)		769 (77.4)	
Sadness**	No	266 (14.7)	**<0.001**	273 (15.0)	**<0.001**	246 (13.6)	**<0.001**	68 (6.8)	**<0.001**
	Yes	1538 (85.3)		1543 (85.0)		1568 (86.4)		926 (93.2)	

*f = Absolute frequency; ^†^% = Relative frequency; defining characteristics of nursing diagnoses; ^‡^Anxiety; ^§^Change Stress syndrome; ^||^Post-trauma syndrome; ^¶^Stress overload; ^**^Chronic sadness, according to the NANDA-I taxonomy^(8)^; p-value < 0.05 - statistically significant

**Table 3-:** Defining characteristics that are predictors of mental health disorders in university students in the post-COVID-19 era (n = 2,349). Viçosa, MG, Brazil, 2024

		Anxiety symptoms		Stress symptoms		Depression symptoms		Post-traumatic stress disorder	
	Categories	ORA* (CI 95% ^†^ )	p-value	ORA* (CI 95% ^†^ )	p-value	ORA* (CI 95% ^†^ )	p-value	ORA* (CI 95% ^†^ )	p-value
**Physical defining characteristics**									
Change in the sleep-wake cycle ^§||^	No ^‡^	-	-	-	-	1		1	
	Yes	-	-	-	-	1.405 (1.054-1.872)	**0.021**	1.437 (1.111-1.858)	**0.006**
Increased sweating ^§^	No ^‡^	1		1		-	**-**	1	
	Yes	1.463 (1.075-1.991)	**0.015**	1.382 (1.016-1.878)	**0.039**	-	**-**	1.355 (1.108-1.658)	**0.003**
Dry mouth ^§^	No ^‡^	1		-	-	1		1	
	Yes	3.312 (2.369-4.630)	**<0.001**	-	-	1.613 (1.209-2.152)	**0.001**	1.551 (1.265-1.901)	**<0.001**
Altered breathing pattern ^§^	No ^‡^	1		1		-	-	1	
	Yes	1.618 (1.170-2.238)	**0.004**	1.877 (1.353-2.604)	**<0.001**	-	-	1.373 (1.097-1.719)	**0.006**
Heart palpitations ^§¶^	No ^‡^	1		1		-	-	1	
	Yes	3.316 (2.379-4.623)	**<0.001**	1.455 (1.058-2.002)	**0.021**	-	-	1.319 (1.062-1.638)	**0.012**
Muscle tension ^§**^	No ^‡^	-	-	1		-	-	-	-
	Yes	-	-	1.444 (1.072-1.943)	**0.016**	-	-	-	-
Headache ^¶^	No ^‡^	-	-	1		-	-	1	
	Yes	-	-	1.298 (0.966-1.744)	0.083	-	-	1.299 (1.054-1.599)	**0.014**
Nausea ^§¶^	No ^‡^	1		1		1		1	
	Yes	2.707 (1.822-4.020)	**<0.001**	1.628 (1.109-2.390)	**0.013**	1.952 (1.397-2.726)	**<0.001**	1.226 (0.933-1.514)	0.058
Tightness in the chest ^§^	No ^‡^	1		-	-	-	-	-	-
	Yes	1.422 (1.041-1.941)	**0.027**	-	-	-	-	-	-
**Behavioral defining characteristics**									
Psychomotor agitation ^§^	No ^‡^	1		1		-	-	1	
	Yes	2.238 (1.614-3.104)	**<0.001**	3.233 (2.312-4.522)	**<0.001**	-	-	1.425 (1.009-2.013)	**0.045**
Easy cry ^§^	No ^‡^	1		1		-	-	1	
	Yes	1.550 (1.170-2.053)	**0.002**	1.813 (1.358-2.420)	**<0.001**	-	-	1.343 (1.076-1.678)	**0.009**
Increased impatience**	No ^‡^	-	-	1		1		-	-
	Yes	-	-	2.225 (1.601-3.093)	**<0.001**	1.319 0.953-1.825)	0.095	-	-
Decreased productivity ^§^	No ^‡^	-	-	-	-	1		-	-
	Yes	-	-	-	-	2.449 (1.827-3.282)	**<0.001**	-	-
Low self-steem ^||^	No ^‡^	1		1		1		1	
	Yes	2.026 (1.476-2.782)	**<0.001**	1.632 (1.189-2.239)	**0.002**	2.205 (1.649-2.949)	**<0.001**	1.696 (1.272-2.260)	**<0.001**
**Cognitive defining characteristics**									
Altered attention ^§¶^	No ^‡^	-	-	1		-	-	-	-
	Yes	-	-	1.649 (1.169-2.325)	**0.004**	-	-	-	-
Frequent forgetfulness ^§^	No ^‡^	1		1		1		1	
	Yes	1.433 (1.061-1.934)	**0.019**	1.644 (1.197-2.257)	**0.002**	1.362 (1.009-1.838)	**0.043**	1.547 (1.189-2.013)	**0.001**
**Emotional defining characteristics**									
Excessive worry ^§||^	Não ^‡^	1		1		1		1	
	Yes	1.728 (1.209-2.471)	**0.003**	2.698 (1.868-3.897)	**<0.001**	1.539 (1.075-2.205)	**0.019**	2.825 (1.764-4.523)	**<0.001**
Frustration ^||^	No ^‡^	1		-	-	1		-	-
	Yes	1.412 (0.991-2.012)	0.056	-	-	2.454 (1.793-3.359)	**<0.001**	-	-
Loneliness ^||^	No ^‡^	-	-	1		1		1	
	Yes	-	-	1.677 (1.237-2.273)	**0.001**	1.822 (1.378-2.407)	**<0.001**	1.420 (1.120-1.801)	**0.004**
Fear ^§||¶^	No ^‡^	1		-	-	1		1	
	Yes	2.036 (1.508-2.748)	**<0.001**	-	-	1.305 (0.972-1.152)	**0.076**	1.677 (1.281-2.196)	**<0.001**
Anger ^||¶^ **	No ^‡^	-	-	1		1		1	
	Yes	-	-	2.213 (1.641-2.986)	**<0.001**	1.923 (1.446-2.557)	**<0.001**	1.597 (1.287-1.983)	**<0.001**
Sadness ^††^	No ^‡^	1		1		1		1	
	Yes	1.487 (1.062-2.082)	**0.021**	1.572 (1.118-2.211)	**0.009**	2.557 (1.871-3.472)	**<0.001**	1.522 (1.091-2.123)	**0.013**

*ORA = Odds Ratio Adjusted; ^†^CI 95% = 95% confidence interval; ^‡^Reference for ORA, defining characteristics of nursing diagnoses; ^§^Anxiety; ^||^Change Stress syndrome; ^¶^Post-trauma syndrome; **Stress overload; ^††^Chronic sadness, according to the NANDA-I taxonomy^(8)^; p-value < 0.05 - statistically significant

## Discussion

This study determined physical, behavioral, cognitive, and emotional defining characteristics of the nursing diagnoses, from the NANDA-I taxonomy^(8)^, Anxiety (00146); Stress Overload (00177); Post-Trauma Syndrome (00141); Change Stress Syndrome (00114), and Chronic Sadness (00137), which were configured as predictors in those university students with mental health disorders in the post-COVID-19 era. Both the defining characteristics of nursing diagnoses observed and mental health disorders were highly prevalent in this population, which makes these findings worrying.

Mental health problems were greatly aggravated during the COVID-19 pandemic and persisted after its end, including in undergraduate students. Research conducted with this population has already reported the prevalence of these problems around the world, in the post-COVID-19 era^(6-7,9)^. However, we found higher prevalence of these mental health disorders in Brazilian students. This denotes the great mental suffering experienced in the post-pandemic era, and the urgent need to establish care policies that value their mental health.

In relation to the physical defining characteristics, increased sweating, altered breathing pattern and heart palpitations emerged as predictors for anxiety, stress and PTSD; dry mouth for anxiety, PTSD and depression; nausea for anxiety, stress and depression; tightness in the chest for anxiety and depression; alteration in the sleep-wake cycle for PTSD and depression; muscle tension for stress; and headache for PTSD.

According to NANDA-I^(8)^, all these physical signs and symptoms, with the exception of headache, are defining characteristics of the diagnosis Anxiety. The diagnosis Change Stress Syndrome also presents as defining characteristics the reference to increased physical symptoms, as well as the diagnosis Stress Overload, that of expressing tension. Heart palpitations and headache are included in the diagnosis of Post-Trauma Syndrome. The diagnosis of Chronic Sadness does not present physical defining characteristics. These findings reaffirm that these defining characteristics are in fact present in people with anxiety, stress and depression; and, in addition, they can lead to the development of these clinical conditions, since they were configured as predictive variables in the present study. In the context of the COVID-19 pandemic, changes in sleep patterns were frequent in university students^(17)^. In the present study, this prevalence was also significant for the four disorders investigated (greater than 80.0%), demonstrating that changes in the sleep-wake cycle remained in the post-COVID-19 era. These data are worrying, since changes in sleep patterns are directly correlated with mental health problems in university students^(12)^. Manifestations such as increased sweating, dry mouth, altered cardiac and respiratory parameters and gastrointestinal changes, such as nausea, can also be present in people with anxiety^(8)^. These manifestations are also investigated in validated instruments to assess anxiety, stress, and depression, such as the DASS-21^(14)^, and in the IES-R to assess PTSD^(15)^. This reaffirms the importance of assessing these characteristics.

In the present study, the prevalence of dry mouth, heart palpitations, and altered breathing patterns were significant in students who manifested anxiety (54.3%, 64.3%, and 64.7%, respectively). And, mainly, cardiac and respiratory parameters were also significant in students who presented PTSD (69% and 71.4%, respectively) and stress (61.8% and 63.8%, respectively). The activation of the hypothalamic-pituitary-adrenal axis and the sympathetic nervous system in stressful situations justify these manifestations^(18)^. In the post-pandemic context, the significant prevalence of PTSD and stress in students reveals the importance of assessing these manifestations in this population.

Muscle tension emerged as a defining characteristic predicting the presence of stress, with a significant prevalence among students in the present study (80.1%). Since the autonomic nervous system innervates virtually all organic systems in the body, activation of the sympathetic response can lead to several physiological changes, such as increased tension and muscle pain^(19)^. In university students, in the context of the pandemic, researchers observed a high prevalence of tension in the muscles of the face and stress levels were significantly higher^(20)^. Likewise, headaches can also occur and be associated with high levels of stress in students^(21)^. In fact, in the present study, this defining characteristic was a predictor of the development of PTSD, with a high prevalence among students (70.2%).

Another physical symptom that was configured as a defining characteristic predicting anxiety and depression was the sensation of tightness in the chest, with a prevalence of 67.2% in students with anxiety and 64.4% with depression. A study conducted with university students found a significant association between the presence of anxiety and the symptom of tightness in the chest or heart; the same did not occur for depression^(22)^. It is important to highlight that this symptom appears as a defining characteristic of the diagnosis Anxiety, in NANDA-I^(8)^. However, due to its high prevalence in students with symptoms of depression and the fact that it emerged as a predictor variable for this condition, it is suggested that the possibility of including this symptom as a defining characteristic of the diagnosis Chronic Sadness be evaluated.

Considering that the physical defining characteristics are more easily perceptible, they can act as warning signs for these conditions. In the university context, for example, when teachers identify the presence of these manifestations in students in the classroom, greater attention should be given to them, in relation to their mental health, so that emotional problems can be identified in their early stages and early interventions can be implemented.

Among the behavioral defining characteristics identified in students, low self-esteem was a predictor of anxiety, stress, depression and PTSD; psychomotor agitation and easy crying for anxiety, stress and PTSD; increased impatience for stress; and decreased productivity for depression. According to NANDA-I^(8)^, low self-esteem is a defining characteristic of the diagnosis of Change Stress Syndrome; psychomotor agitation, easy crying and decreased productivity are characteristics of the diagnosis of Anxiety; and increased impatience is a defining characteristic of Stress Overload.

Low self-esteem can be understood as the negative perception of self-worth, self-acceptance, self-respect, competence and attitude towards oneself^(8)^. In the university context, low self-esteem is an influencing factor for higher levels of depression^(23)^, in addition to significantly compromising academic performance^(24)^. This fact was aggravated by the pandemic and continues in the post-pandemic context, when we observe the significant prevalence of low self-esteem, which ranged from 84.9% to 90.1% in students with mental health disorders in this study. Therefore, assessing students’ self-esteem can contribute to establishing diagnoses related to mental health more accurately and, consequently, to establishing more effective interventions.

Psychomotor agitation and easy crying were also highly prevalent (approximately 92.0% and 75.0%, respectively) in students with anxiety, stress and PTSD. The DASS-21^(14)^ presents, in the anxiety construct, the presence of tremors, for example in the hands and, in the stress construct, difficulties in calming down and relaxing, and agitation. These symptoms are related to psychomotor agitation, which reinforces the importance of their assessment in people with symptoms of anxiety, stress and in post-traumatic situations. Easy crying, which emerged as a predictor for these conditions, is also presented by the DASS-21^(14)^, but indirectly as “I felt I was a little too emotional/sensitive” in the depression construct. The need to detect these defining characteristics in students, who were strongly impacted psychologically by the COVID-19 pandemic, is reaffirmed. Increased impatience was also significant in students who presented symptoms of stress (90.7%) in the post-pandemic context. This is also a symptom assessed by the IES-R in people with PTSD (“I felt irritable and angry”)^(15)^. The various consequences of the pandemic on the way of teaching led students to develop feelings of impatience, annoyance, and hostility^(25)^, and this may predispose them to the development of stress, as evidenced by the present study. Given this, it is important to work with coping and stress relief strategies with this population, so that these negative feelings do not impact the student’s academic performance.

Another frequent symptom in the students in this study and which was configured as a predictor for depression was decreased productivity (85.8%). It is noteworthy that the lack of desire to carry out daily activities is a characteristic of depression. In the university context, this lack of desire may reflect in decreased productivity, with a consequent impact on academic performance. However, in NANDA-I, the Chronic Sadness diagnosis does not present this defining characteristic^(8)^. Due to the high prevalence evidenced by this study and its direct relationship with depression, the possibility of inclusion is suggested.

Among the cognitive signs and symptoms, frequent forgetfulness was a predictor for anxiety, stress, PTSD and depression; and altered attention for stress and PTSD. According to NANDA-I, forgetfulness is configured as a defining characteristic of the Anxiety diagnosis; and altered attention is a defining characteristic of Anxiety and Post-Trauma Syndrome. Furthermore, IES-R presents concentration problems as characteristics of PTSD^(15)^, reinforcing the need to evaluate them.

During the COVID-19 pandemic, difficulty concentrating was frequent among university students, with a prevalence of around 89%^(17)^. Cognitive problems persisted until the post-pandemic era, a fact confirmed by the present study, which found a prevalence of frequent forgetfulness greater than 80% in all constructs evaluated, and of altered attention of 88.9% and 91.2% in students with stress and PTSD, respectively. These findings are important because the presence of these defining characteristics can compromise students’ academic performance, reduce motivation for studies and, ultimately, contribute to school dropout.

Among the defining emotional characteristics, excessive worry and sadness were predictors of anxiety, stress, PTSD and depression; loneliness and anger for stress, PTSD and depression; fear for anxiety and PTSD; and frustration for depression.

In the present study, a high prevalence of feelings of worry (>93.0% in all constructs) and sadness (≥85.0% in all constructs) was observed in students. In a study conducted with 195 university students to understand the effects of the pandemic on their mental health, it was found that concern about academic performance and excessive and depressive thoughts were also present in this population; in addition, these thoughts contributed to the occurrence of suicidal thoughts^(17)^. Given these findings, it is clear how these feelings still persist in the post-pandemic context, and that, when exacerbated, they can compromise the mental health of students and lead them to risky behaviors. Therefore, when assessing the mental health of students, it is suggested to observe the presence of these feelings when listing all the nursing diagnoses studied here.

Feelings of loneliness^(17)^ and anger^(26)^, which were present in students during the pandemic, were also highly prevalent in the post-COVID-19 era. In fact, more than 75.0% and 69.0% of the students in this study presented these symptoms, respectively. According to NANDA-I, these feelings are present in the diagnoses of Change Stress Syndrome, Post-Trauma Syndrome and Stress Overload^(8)^. However, in the present study, they also emerged as predictors of depression in undergraduate students, so we suggest evaluating these feelings also in cases of sadness.

Fear, which was also reported by university students in the pandemic context^(17)^, was very frequently present in the post-COVID-19 era, as evidenced by our study, especially in students with anxiety (83.1%) and PTSD (88.5%). Fear can influence multiple aspects of people’s lives and health^(27)^. During the pandemic, this reaction triggered serious pathophysiological, social, behavioral, and mental consequences^(27)^. In fact, a study showed that the higher prevalence of fear of COVID-19 was related to worse sleep quality, worse health perception, sadness, greater stress, depressive symptoms, and suicidal ideation^(27)^. Thus, understanding and mitigating fear in the university student population is a major concern and a focal point for interventions^(28)^. It is also noteworthy that according to NANDA-I^(8)^, this feeling is present in the diagnoses Anxiety, Change Stress Syndrome, and Post-Trauma Syndrome, demonstrating that the results found are in accordance with the taxonomy.

Finally, frustration was another significant symptom in the present study, with a prevalence of 91.5% in students with depression. During the pandemic, students expressed frustrations regarding remote classes; access to technologies; the interruption of research and extension activities; and family, social, emotional, behavioral, and financial aspects of life^(29)^. And, with the findings presented here, it is clear that this feeling still persists in the post-COVID-19 era. Ensuring, therefore, that students’ frustrations and barriers to success are recognized and considered can help prevent dropout from higher education^(29)^.

In view of the results of this investigation, we suggest that the physical, behavioral, cognitive and emotional defining characteristics that emerged as predictors of anxiety, stress, PTSD and depression in undergraduate students in the post-COVID-19 era should be further studied as possible defining characteristics of the respective diagnoses Anxiety; Change Stress syndrome; Post-trauma syndrome; Stress Overload; Chronic Sadness. They can provide more diagnostic clues and, consequently, facilitate the implementation of effective interventions for this population.

This study has limitations that should be highlighted. First, the online data collection prevented the calculation of an accurate response rate; in addition, students without internet access were unable to participate. In future research, face-to-face data collection would avoid selection bias. Second, self-report questionnaires were used to assess symptoms related to mental health disorders, as well as the presence of defining characteristics of the nursing diagnoses studied. For future studies, it is suggested that clinical diagnoses be performed by physicians and that the defining characteristics be assessed by nurses, so that these data are not based solely on participant observation. Third, this study was limited to only one region of the country, which may restrict, in part, the generalization of the results to other regions or countries. However, the significant sample size, from 15 campuses (in different cities) and 94 undergraduate courses, increased the reliability and validity of the comparisons made. In addition, associations with ORA lower than 2.0 should be interpreted with caution, since they may produce a false positive association^(30)^. It is also suggested that the academic semester be considered in the statistical analysis, since students from different semesters may present different characteristics. Finally, we emphasize that the most current version of NANDA-I was not used, since it had not been released at the time of data collection. However, the researchers observed that the defining characteristics investigated in this study were not included.

Despite its limitations, this study advances scientific knowledge, as it is among the first to identify the prevalence of mental health disorders among undergraduate students in the post-COVID-19 era in Brazil, especially in the nursing field, since it highlighted defining characteristics of nursing diagnoses that were configured as predictors of anxiety, stress, depression, and PTSD. These findings can help nurses improve the assessment of mental health disorders in the university student population, as well as to implement effective interventions to promote mental health.

In addition, it is noteworthy that the most current version of NANDA-I, released in April 2024^(31)^, does not include the diagnoses Change Stress Syndrome, Stress Overload, and Chronic Sadness. Considering the high prevalence of the defining characteristics of these diagnoses in a significant sample of undergraduate students, which were predictors of anxiety, stress, depression, and PTSD, this study validates their occurrence. Therefore, it is suggested that these diagnoses, with their respective defining characteristics, be reinserted into the NANDA-I taxonomy.

## Conclusion

This study highlighted physical, behavioral, cognitive and emotional defining characteristics present in the nursing diagnoses Anxiety; Change Stress Syndrome; Post-Trauma Syndrome; Stress Overload; Chronic Sadness, according to NANDA-I, which were configured as predictors for the development of mental health disorders in university students in the post-COVID-19 era, such as: alteration in the sleep-wake cycle; increased sweating; dry mouth; altered breathing pattern; heart palpitations; muscle tension; headache; nausea; chest tightness; psychomotor agitation; easy crying; increased impatience; decreased productivity; low self-esteem; altered attention; frequent forgetfulness; excessive worry; frustration; loneliness; fear; anger; and sadness.

## Data Availability

The dataset of this article is available on the RLAE page in the SciELO Data repository, at the link https://doi.org/10.48331/scielodata.D4GINI

## References

[1] World Health Organization (2022). World mental health report: transforming mental health for all.

[2] Collaborators GBD 2019 Mental Disorders (2022). Global, regional, and national burden of 12 mental disorders in 204 countries and territories, 1990–2019: a systematic analysis for the Global Burden of Disease Study 2019. Lancet Psychiatry.

[3] Kang H. K., Rhodes C., Rivers E., Thornton C. P., Rodney T. (2021). Prevalence of Mental Health Disorders Among Undergraduate University Students in the United States: A Review. J Psychosoc Nurs Ment Health Serv.

[4] Li W., Zha Z., Chen D., Peng Y., Lu Z. (2022). Prevalence and associated factors of depression and anxiety symptoms among college students: a systematic review and meta-analysis. J Child Psychol Psychiatry.

[5] Lopes A. R., Nihei O. K. (2021). Depression, anxiety and stress symptoms in Brazilian university students during the COVID-19 pandemic: Predictors and association with life satisfaction, psychological well-being and coping strategies. PLoS One.

[6] Wang X., Zhang N., Pu C., Li Y., Chen H., Li M. (2022). Anxiety, Depression, and TEPT among College Students in the Post-COVID-19 Era: A Cross-Sectional Study. Brain Sci.

[7] Martínez-Líbano J., Torres-Vallejos J., Oyanedel J. C., González-Campusano N., Calderón-Herrera G., Yeomans-Cabrera M. M. (2023). Prevalence and variables associated with depression, anxiety, and stress among Chilean higher education students, post-pandemic. Front Psychiatry.

[8] Herdman T. H., Kamitsuru S., Lopes C. T. (2021). Diagnósticos de Enfermagem da NANDA: definições e classificações 2021-2023.

[9] Estacio R. D., Subida M. D., Bagaforo V., Abuyuan C., Dionisio N. Y., Letso S. C. (2023). The mental health of college students in the transition of post-pandemic era in Pampanga, Philippines: an input for psychoeducation programme. Asia Pac J Couns Psy.

[10] Estrada-Araoz E. G., Quispe J. A. B., Córdova-Rojas L. M., Chayña E. T., Coaquira H. M., Tomanguilla J. H. (2023). Mental Health of University Students When Returning to Face-to-Face Classes: A Cross-Sectional Study. Behav Sci.

[11] Farfán-Latorre M., Estrada-Araoz E. G., Lavilla-Condori W. G., Ulloa-Gallardo N. J., Calcina-Álvarez D. A., Meza-Orue L. A. (2023). Mental Health in the Post-Pandemic Period: Depression, Anxiety, and Stress in Peruvian University Students upon Return to Face-to-Face Classes. Sustainability.

[12] Chautrakarn S., Jaiprom E., Ong-Artborirak P. (2024). Mental health and sleep in the post-COVID-19 era among Thai undergraduate students. Sci Rep.

[13] Elm E. von, Altman D. G., Egger M., Pocock S. J., Gotzsche P. C., Vandenbroucke J. P. (2007). The Strengthening the Reporting of Observational Studies in Epidemiology (STROBE) Statement: guidelines for reporting observational studies. Lancet.

[14] Vignola R. C., Tucci A. M. (2014). Adaptation and validation of the depression, anxiety and stress scale (DASS) to Brazilian Portuguese. J Affect Disord.

[15] Caiuby A. V. S., Lacerda S. S., Quintana M. I., Torii T. S., Andreoli S. B. (2012). Cross-cultural adaptation of the Brazilian version of the Impact of Events Scale-Revised (IES-R). Cad Saude Publica.

[16] Tavakol M., Dennick R. (2011). Making sense of Cronbach’s alpha. Int J Med Educ.

[17] Son C., Hegde S., Smith A., Wang X., Sasangohar F. (2020). Effects of COVID-19 on College Students’ Mental Health in the United States: Interview Survey Study. J Med Internet Res.

[18] Nicolaides N. C., Kyratzi E., Lamprokostopoulou A., Chrousos G. P., Charmandari E. (2015). Stress, the Stress System and the Role of Glucocorticoids. Neuroimmunomodulation.

[19] Shuda Q., Bougoulias M. E., Kass R. (2020). Effect of nature exposure on perceived and physiologic stress: A systematic review. Complement Ther Med.

[20] Saczuk K., Lapinska B., Wawrzynkiewicz A., Witkowska A., Arbildo-Vega H. I., Domarecka M. (2022). Temporomandibular Disorders, Bruxism, Perceived Stress, and Coping Strategies among Medical University Students in Times of Social Isolation during Outbreak of COVID-19 Pandemic. Healthcare.

[21] Francisco J., Golshan F., Morrison T. G., Mickleborough M. (2023). Stress and headaches in university students during the COVID-19 pandemic. PLoS One.

[22] Soares L. F. F., Coelho L. M., Moreno A., Almeida D. A. F., Haddad M. F. (2020). Anxiety and depression associated with pain and discomfort of temporomandibular disorders. BrJP.

[23] Azmi F. M., Khan H. N., Azmi A. M., Yaswi A., Jakovljevic M. (2022). Prevalence of COVID-19 Pandemic, Self-Esteem and Its Effect on Depression Among University Students in Saudi Arabia. Front Public Health.

[24] Yu W., Qian Y., Abbey C., Wang H., Rozelle S., Stoffel L. A. (2022). The Role of Self-Esteem in the Academic Performance of Rural Students in China. Int J Environ Res Public Health.

[25] Olawale B. E., Mutongoza B. H., Adu E. O., Omodan B. I. (2021). COVID-19 induced psychosocial challenges in South African higher education: Experiences of staff and students at two rural universities. Res Soc Sci Technol.

[26] Panchal U., Pablo G. S., Franco M., Moreno C., Parellada M., Arango C. (2023). The impact of COVID-19 lockdown on child and adolescent mental health: systematic review. Eur Child Adolesc Psychiatry.

[27] Meller F. O., Schäfer A. A., Quadra M. R., Demenech L. M., Paludo S. D. S., Silva P. A. (2022). Fear of COVID-19 and health-related outcomes: results from two Brazilian population-based studies. Psychiatry Res.

[28] Zolotov Y., Reznik A., Bender S., Isralowitz R. (2022). COVID-19 Fear, Mental Health, and Substance Use Among Israeli University Students. Int J Ment Health Addict.

[29] Hagedorn R. L., Wattick R. A., Olfert M. D. (2022). "My Entire World Stopped": College Students’ Psychosocial and Academic Frustrations during the COVID-19 Pandemic. Appl Res Qual Life.

[30] Nicolich M. J., Gamble J. F., Moldoveanu A. M. (2011). Advanced Topics in Environmental Health and Air Pollution Case Studies.

[31] Herdman T. H., Kamitsuru S., Lopes C. T. (2024). NANDA International Nursing Diagnoses: Definitions and Classification, 2024-2026.

