# Supplementary material for: Defining characteristics predicting mental health disorders among undergraduate students in the post-COVID-19 era: a multicenter cross-sectional study
Source: Rev Lat Am Enfermagem. 2025 Oct 27;33:e4671. doi: 10.1590/1518-8345.7741.4671 (PMC12562638; doi:10.1590/1518-8345.7741.4671)
Supplement: Translation

# Translation: Características definidoras preditoras de desordens de saúde mental entre estudantes de graduação na era pós-COVID-19: estudo transversal multicêntrico*

**DOI:** 10.1590/1518-8345.7741.4672

**Authors:** Caroline de Castro Moura, Luana Vieira Toledo, Gabriela Tavares Boscarol, Rafael Lopes Chaves, Denismar Alves Nogueira, Carine Alves da Costa Marinho, Bruna de Oliveira Alves, Bárbara Guimarães Lourenço, Érika de Cássia Lopes Chaves, Tânia Couto Machado Chianca

**Affiliations:** 1 Universidade Federal de Viçosa, Departamento de Medicina e Enfermagem, Viçosa, MG, Brasil.; 2 Universidade Federal de Minas Gerais, Escola de Enfermagem, Belo Horizonte, MG, Brasil.; 3 Bolsista da Coordenação de Aperfeiçoamento de Pessoal de Nível Superior (CAPES), Brasil.; 4 Universidade Professor Edson Antônio Velano, Curso de Psicologia, Alfenas, MG, Brasil.; 5 Bolsista da Fundação de Amparo à Pesquisa do Estado de Minas Gerais (FAPEMIG), Brasil.; 6 Universidade Federal de Alfenas, Departamento de Estatística, Alfenas, MG, Brasil.; 7 Universidade Federal de Alfenas, Escola de Enfermagem, Alfenas, MG, Brasil.

**Keywords:** Sinais e Sintomas, Saúde Mental, Universidades, Estudantes, COVID-19, Enfermagem

## Abstract

avaliar características definidoras de diagnósticos de enfermagem que se configuram como preditoras de desordens de saúde mental entre estudantes de graduação na era pós-COVID-19.

estudo transversal, multicêntrico, realizado com estudantes de graduação de quatro universidades públicas do estado de Minas Gerais. Os dados foram coletados por meio de questionário estruturado, *online*, com variáveis sociodemográficas e clínicas. Investigou-se, ainda, por meio de autorrelato, a presença de características definidoras incluídas nos Diagnósticos de Enfermagem de Ansiedade, Síndrome do Estresse por Mudança; Síndrome Pós-trauma; Sobrecarga de Estresse e Tristeza Crônica, presentes na taxonomia da NANDA-I. Foram utilizadas, também, a *Depression, Anxiety and Stress Scale* e a *Impact of Events Scale-Revised*. Análise de regressão logística multivariada foi realizada para determinar as características definidoras que se configuraram como preditoras das desordens de saúde mental. *Odds ratios* ajustados foram estimados, juntamente com os intervalos de confiança de 95%, a 5% de significância.

participaram do estudo 2.349 estudantes de graduação. Foi investigada a presença de 24 características definidoras físicas, comportamentais, cognitivas e emocionais dos diagnósticos de enfermagem em questão. Destas, 13 emergiram como preditoras para os sintomas de ansiedade; 15 para os sintomas de estresse; 11 para os sintomas de depressão; e 15 para o transtorno do estresse pós-traumático.

evidenciaram-se características definidoras físicas, comportamentais, cognitivas e emocionais de diagnósticos de enfermagem da NANDA-I que se configuraram como preditoras para o desenvolvimento de desordens de saúde mental em universitários na era pós-COVID-19.

## Destaques

**(1)** Desordens de saúde mental são prevalentes em universitários na era pós-COVID-19. **(2)** Diagnósticos de enfermagem relacionados à saúde mental são prevalentes em estudantes. **(3)** Características definidoras emergiram como preditoras para desordens de saúde mental. **(4)** É necessário implementar intervenções para promover a saúde mental nessa população.

## Introdução

Segundo a *World Health Organization*, a saúde mental é definida como “um estado de bem-estar no qual o indivíduo percebe suas próprias habilidades, consegue lidar com os estresses cotidianos, trabalha produtivamente e é capaz de contribuir para sua comunidade”^(1)^. As desordens relacionadas a ela representam um grande problema de saúde pública a nível mundial e surgem quando a pessoa não consegue adaptar-se às mudanças que ocorrem em sua vida, gerando grande sofrimento^(1)^.

Em todo o mundo, uma em cada oito pessoas vivem com desordens de saúde mental^(1)^. Os resultados do *Global Burden of Diseases* apontaram que entre os anos de 1990 e 2019, o número global de anos de vida ajustados por incapacidade devido a transtornos mentais aumentou de 80,8 milhões para 125,3 milhões, com grande ônus para as pessoas em todo o mundo^(2)^.

Os estudantes de graduação representam uma população vulnerável ao desenvolvimento de desordens de saúde mental^(3)^, e a pandemia da COVID-19 as agravou ainda mais^(4-5)^, pois dentre os seus inúmeros impactos negativos, desencadeou uma crise global na saúde mental das pessoas^(1)^. De fato, ansiedade, estresse, depressão e transtorno do estresse pós-traumático (TEPT), permaneceram prevalentes na era pós-COVID-19^(6-7)^.

No contexto da enfermagem, a taxonomia da NANDA-I^(8)^ apresenta alguns diagnósticos relacionados à saúde mental que podem estar presentes na população universitária, e provavelmente foram intensificados devido ao contexto pandêmico. Dentre eles, destacam-se a Ansiedade (00146); Sobrecarga de Estresse (00177); Síndrome Pós-trauma (00141); Síndrome do Estresse por Mudança (00114) e Tristeza Crônica (00137). Atentar-se para as características definidoras e fatores relacionados que podem desencadear esses problemas, a fim de implementar medidas efetivas para solucioná-los e preveni-los, faz-se essencial nesse contexto de crise da saúde mental.

Globalmente, estudos já avaliaram desordens de saúde mental em estudantes universitários na era pós-COVID-19^(6-7,9-12)^. Como exemplo, estudo conduzido na China^(6)^ identificou que fatores associados a mudanças sociais, familiares e econômicas impostas pela pandemia aumentaram o risco de sintomas psicológicos em estudantes universitários. Outro estudo^(7)^ realizado no Chile identificou, além de altas prevalências de depressão, ansiedade e estresse, que ser do sexo feminino, pertencer a minorias sexuais e uso de psicofármacos parecem ter impacto na suscetibilidade a problemas de saúde mental. Ademais, um estudo conduzido na Tailândia^(12)^ encontrou que condições médicas, relacionamentos ruins com familiares, amigos ou outras pessoas, ter problemas enquanto estudava na universidade e o impacto autopercebido da COVID-19 na vida do aluno foram aspectos associados a problemas de saúde mental.

Destaca-se que grande parte dos estudos envolveram, principalmente, o rastreamento do estado psicológico e sua associação com fatores demográficos, clínicos e acadêmicos^(6-7,9-12)^. No entanto, as investigações no Brasil são escassas e até o presente momento não foram encontrados, a nível nacional ou internacional, estudos de revisão sistemática ou de escopo, ou que avaliaram a presença de características definidoras de diagnósticos de enfermagem em estudantes de graduação na era pós-COVID-19, e as estimou como preditoras de ansiedade, estresse, depressão e TEPT. Avaliar essas características em uma população vulnerável ao desenvolvimento de desordens de saúde mental e que vivenciou uma grande crise sanitária pode ser um passo importante no desenvolvimento de estratégias institucionais de cuidado para esse público. Nesse sentido, o presente estudo objetivou avaliar características definidoras de diagnósticos de enfermagem que se configuram como preditoras de desordens de saúde mental entre estudantes de graduação na era pós-COVID-19.

## Método

### Delineamento do estudo

Estudo transversal, multicêntrico, realizado com estudantes universitários de graduação e reportado de acordo com as recomendações do *Strengthening the Reporting of Observational Studies in Epidemiology* ^(13)^.

### Local e período

O estudo foi realizado em quatro universidades públicas do estado de Minas Gerais, Brasil, entre janeiro de 2023 a abril de 2024. A universidade 1 possui 79 cursos de graduação distribuídos em cinco *campi* nas regiões Metropolitana e Norte do estado de Minas Gerais. A universidade 2 possui 56 cursos de graduação, em três *campi* na região Metropolitana. Já a universidade 3 conta com 67 cursos de graduação em três *campi* na Zona da Mata, região Metropolitana e Alto Paranaíba/Triângulo Mineiro. Por fim, a universidade 4 é composta por 38 cursos de graduação em quatro *campi*, localizados no Sul e Sudoeste do estado.

### População e definição da amostra

A população foi constituída por 67.110 estudantes universitários de graduação, sendo 33.900 na universidade 1, 13.110 na universidade 2, 14.000 na universidade 3, e 6.100 na universidade 4. Para o cálculo da amostra, foi utilizada uma estimativa conservadora de prevalência de 50%, já que a prevalência de desordens de saúde mental na era pós-COVID-19 no Brasil era desconhecida no momento do planejamento do estudo. Foi utilizada a fórmula n = (z^2^.p(1-p)/e^2^)/1+(z^2^.p(1-p)/e^2^N), em que: z = escore z (para grau de confiança de 95%, z = 1,96); p = prevalência de casos com a característica estudada; e = margem de erro; N = tamanho da população. Ao considerar a população de 67.110 estudantes e margem de erro de 2%, o tamanho da amostra mínimo estimado para o presente estudo foi de 2.319 estudantes.

### Critérios de seleção

Estudantes com 18 anos ou mais, que estavam regularmente matriculados em qualquer período dos cursos de graduação e que tinham disponibilidade de horário para responder aos instrumentos de coleta de dados foram recrutados. Foram excluídos do estudo aqueles que não tinham acesso à *internet* no momento da coleta de dados e que não completaram todo o inquérito.

### Variáveis do estudo

A presença ou ausência de sintomas de ansiedade, estresse, depressão e TEPT foram consideradas variáveis dependentes. As características definidoras dos diagnósticos de enfermagem estudados foram consideradas explicativas ou preditoras. Características sociodemográficas e clínicas foram consideradas covariáveis.

### Instrumentos utilizados para a coleta das informações

As seguintes características sociodemográficas e clínicas foram coletadas por meio de um questionário estruturado *online*: sexo biológico; cor da pele; situação conjugal; moradia; renda familiar (em salários-mínimos do Brasil - no momento da coleta de dados: R$1.320,00); atividade laboral; assistência estudantil; bolsa estudantil (de ensino, pesquisa ou extensão); diagnóstico clínico de desordens de saúde mental; uso de psicofármacos; acompanhamento com psiquiatra e psicólogo. Além disso, foi investigada, por meio de autorrelato, a presença de características definidoras dos seguintes diagnósticos de enfermagem, de acordo com a taxonomia NANDA-I^(8)^:

1) Ansiedade: agitação psicomotora; choro fácil; insegurança; alteração no ciclo sono-vigília; produtividade diminuída; aperto no peito; boca seca; diarreia; tensão muscular; palpitação cardíaca; náusea; padrão respiratório alterado; rubor facial; transpiração aumentada; atenção alterada; esquecimento; preocupação; medo.
2) Síndrome do estresse por mudança: baixa autoestima; raiva; medo; preocupação; frustração; alteração no ciclo-vigília; solidão.
3) Sobrecarga de estresse: raiva; impaciência aumentada; tensão muscular.
4) Síndrome pós-trauma: atenção alterada; cefaleia; raiva; medo; palpitação cardíaca.
5) Tristeza crônica: tristeza.

Essas características definidoras foram selecionadas pois foram observadas com mais frequência em estudantes de graduação, de acordo com a prática clínica de seis pesquisadores da equipe, que são enfermeiros e docentes universitários. Além disso, algumas dessas características definidoras também estão presentes na *Depression, Anxiety, and Stress Scale* (DASS-21)^(14)^ e na *Impact of Events Scale-Revised* (IES-R)^(15)^.

Para mensurar os sintomas de ansiedade, estresse e depressão foi utilizada a DASS-21^(14)^. A escala conta com 21 questões, em forma de autorrelato, sendo dividida em três subescalas que avaliam sintomas percebidos na última semana, por meio de sete perguntas, com quatro opções de respostas (0 = não se aplicou de maneira alguma a 3 = aplicou-se muito ou na maioria do tempo). O somatório de cada subescala, multiplicado por dois, fornece o escore total de cada construto^(14)^. A escala foi traduzida e validada para a versão brasileira e possui adequadas propriedades psicométricas^(14)^. Na presente amostra, o alfa de Cronbach global foi de 0.944 (para as subescalas ansiedade, estresse e depressão foi 0.867, 0.886 e 0.901, respectivamente)^(16)^.

Para avaliar o TEPT foi utilizada a IES-R^(15)^. A IES-R é autoaplicável e apresenta 22 itens subdivididos em três subescalas de sintomas: evitação, intrusão e hiperestimulação, que consideram os últimos sete dias. Cada item compreende cinco opções de resposta (0- nenhum pouco a 4- extremamente). O resultado geral é a soma das médias de cada subescala. Valor maior que 5,6 indica a presença de TEPT^(15)^. O instrumento foi validado e adaptado para a versão brasileira^(15)^, e na presente amostra, o alfa de Cronbach foi 0.961^(16)^.

### Coleta de dados

O estudo foi conduzido de forma *online*, via plataforma *Google Forms*, durante três semestres, a fim de atingir o número de participantes estimado pelo cálculo amostral. O estudo foi divulgado nos canais de comunicação oficial das universidades, nos *e-mails* institucionais e por meio de entrega de panfletos. Fornecemos a todos os potenciais participantes informações detalhadas sobre os objetivos da pesquisa para que pudessem tomar decisões informadas sobre participar ou não. Aqueles que concordaram assinaram o termo de consentimento livre e esclarecido em formato digital. Em seguida, responderam, por meio de autorrelato, aos instrumentos de coleta de dados. Os dados foram armazenados em formato eletrônico protegido por senha.

### Tratamento e análise dos dados

A partir das respostas obtidas pelo *Google Forms*, foi gerado um banco de dados no *software Microsoft Office Excel*, versão 2021. Os dados foram importados para o *software* IBM SPSS *Statistics for Windows*, versão 26 (IBM Corp., Armonk, NY, USA) para análises. Frequências relativas e absolutas, média e desvio-padrão resumiram as variáveis estudadas. Análise bivariada, por meio do teste de Qui-quadrado de Pearson, foi utilizada para avaliar a associação entre as características definidoras dos diagnósticos de enfermagem estudados e os sintomas de ansiedade, estresse, depressão e TEPT avaliados nas escalas utilizadas.

Para a análise de regressão múltipla, as multicolinearidades das variáveis também foram avaliadas. O teste VIF para colinearidade apresentou valores próximos a 1, não apresentando problema de multicolinearidade. A presença ou ausência de sintomas de ansiedade, estresse, depressão e TEPT foram consideradas variáveis dependentes. As variáveis explicativas (características definidoras dos diagnósticos de enfermagem estudados) foram inseridas pelo método *Backward* no modelo de regressão logística múltipla. Os *odds ratios* ajustados (ORA) foram estimados, juntamente com os seus intervalos de confiança de 95% (IC 95), os quais foram usados para determinar aquelas características definidoras que poderiam se configurar como preditoras dos sintomas de ansiedade, estresse, depressão e ao TEPT. A associação das variáveis foi considerada estatisticamente significativa para p<0,05.

As variáveis sociodemográficas e clínicas foram colocadas no grupo de variáveis do modelo de regressão logística múltipla; mas, como elas não foram estatisticamente significativas (p>0,05), optou-se por manter somente as características definidoras dos diagnósticos de enfermagem em questão.

## Aspectos éticos

As normas éticas para pesquisas que envolvem seres humanos foram rigorosamente seguidas. O estudo foi aprovado por Comitê de Ética em Pesquisa sob os pareceres número 5.700.107/ CAAE: 63318222.0.0000.5153. Todos os estudantes que concordaram participar do estudo assinaram o Termo de Consentimento Livre e Esclarecido.

## Resultados

Um total de 2.409 estudantes de graduação foram avaliados, dos quais 97,5% (2.349) completaram o estudo. 2,5% (60) foram excluídos da análise, pois eram estudantes de pós-graduação (48); recusaram a participar do estudo (7); não responderam ao inquérito de forma completa (3); ou não tinham 18 anos (2).

A idade dos participantes variou de 18 a 62 anos, com média de 23,4 anos (desvio padrão: 5,4). 48,6% (1.141) estavam matriculados em cursos da área de ciências biológicas e da saúde; 24,2% (569) em ciências humanas, artes e letras; 21,3% (501) em ciências exatas e tecnológicas e 5,9% (138) em ciências agrárias. A descrição das características sociodemográficas e clínicas está na Tabela 1.

**Tabela 1-:** Características sociodemográficas e clínicas dos estudantes universitários de graduação (n = 2.349). Viçosa, MG, Brasil, 2024

**Variáveis**	f*	**(%** ^†^ **)**
**Sexo biológico**		
Masculino	572	24,4
Feminino	1.777	75,6
**Cor da pele**		
Branco	1.353	57,6
Amarelo	27	1,1
Pardo	676	28,8
Preto	287	12,2
Indígena	6	0,3
**Situação conjugal**		
Solteiro	2.203	93,8
Casado/união estável	132	5,6
Viúvo	1	0,1
Divorciado	13	0,6
**Moradia**		
Sozinho	410	17,5
Com a família	942	40,1
Pensão	58	2,5
República	794	33,8
Alojamento estudantil	145	6,2
**Renda familiar em salários-mínimos** ^‡^		
Até 3	1.380	58,7
4 ou mais	969	41,3
**Atividade laboral no momento**	606	25,8
**Assistência estudantil no mento**	621	26,4
**Bolsa estudantil no momento**	476	20,3
**Diagnóstico clínico de desordens de saúde mental**	1.337	56,9
**Uso de psicofármaco**	956	40,7
**Acompanhamento com psiquiatra**		
Não	1.638	69,7
Já fazia antes da pandemia	302	12,9
Começou a fazer durante/após a pandemia	409	17,4
**Acompanhamento com psicólogo**		
Não	1.254	53,4
Já fazia antes da pandemia	461	19,6
Começou a fazer durante/após a pandemia	634	27,0

*f = Frequência absoluta; ^†^% = Frequência relativa; ^‡^Valor do salário-mínimo no momento da coleta de dados: R$1.320,00, 2023

Dentre os 2.349 estudantes que participaram do estudo, 76,8% (1.804) possuíam sintomas de ansiedade; 77,3% (1.816) sintomas de estresse; 77,2% (1.814) sintomas de depressão; e 42,2% (994) TEPT, que foram mensurados pelas escalas empregadas.

A análise bivariada demonstrou que todas as características definidoras dos diagnósticos de enfermagem, selecionadas para mensuração e relatadas pelos estudantes, associaram-se de forma estatisticamente significativa aos sintomas de ansiedade, estresse, depressão e TEPT, na era pós-COVID-19 (Tabela 2).

A Tabela 3 apresenta as características definidoras que se configuraram como preditoras das desordens de saúde mental entre estudantes universitários de graduação a partir dos modelos de regressão logística multivariada.

**Tabela 2-:** Associação entre características definidoras referidas pelos estudantes universitários de graduação e a presença de sintomas de ansiedade, estresse, depressão e transtorno do estresse pós-traumático, na era pós-COVID-19 (n = 2.349). Viçosa, MG, Brasil, 2024

		Sintomas de ansiedade f* (% ^†^ )	Valor p	Sintomas de estresse f* (% ^†^ )	Valor p	Sintomas de depressão f* (% ^†^ )	Valor p	Transtorno do estresse pós-traumático f* (% ^†^ )	Valor p
**Características definidoras físicas**									
Alteração no ciclo sono-vigília ^‡§^	Não	329 (18,2)	**<0,001**	326 (18,0)	**<0,001**	335 (18,5)	**<0,001**	130 (13,1)	**<0,001**
	Sim	1475 (81,8)		1490 (82,0)		1479 (81,5)		864 (86,9)	
Transpiração aumentada ^‡^	Não	900 (49,9)	**<0,001**	917 (50,5)	**<0,001**	940 (51,8)	**<0,001**	433 (43,6)	**<0,001**
	Sim	904 (50,1)		899 (49,5)		874 (48,2)		561 (56,4)	
Boca seca ^‡^	Não	825 (45,7)	**<0,001**	871 (48,0)	**<0,001**	882 (48,6)	**<0,001**	391 (39,3)	**<0,001**
	Sim	979 (54,3)		945 (52,0)		932 (51,4)		603 (60,7)	
Padrão respiratório alterado ^‡^	Não	636 (35,3)	**<0,001**	657 (36,2)	**<0,001**	701 (38,6)	**<0,001**	284 (28,6)	**<0,001**
	Sim	1168 (64,7)		1159 (63,8)		1113 (61,4)		710 (71,4)	
Palpitações cardíacas ^‡||^	Não	644 (35,7)	**<0,001**	694 (38,2)	**<0,001**	720 (39,7)	**<0,001**	308 (31,0)	**<0,001**
	Sim	1160 (64,3)		1122 (61,8)		1094 (60,3)		686 (69,0)	
Tensão muscular ^‡¶^	Não	373 (20,8)	**<0,001**	362 (19,9)	**<0,001**	408 (22,5)	**<0,001**	168 (16,9)	**<0,001**
	Sim	1431 (79,3)		1454 (80,1)		1406 (77,5)		826 (83,1)	
Cefaleia ^||^	Não	641 (35,5)	**<0,001**	646 (35,6)	**<0,001**	6607 (36,8)	**<0,001**	296 (29,8)	**<0,001**
	Sim	1163 (64,5)		1170 (64,4)		1147 (63,2)		698 (70,2)	
Náusea ^‡||^	Não	1080 (59,9)	**<0,001**	1101 (60,6)	**<0,001**	1117 (61,6)	**<0,001**	545 (54,8)	**<0,001**
	Sim	724 (40,1)		715 (39,4)		697 (38,4)		449 (45,2)	
Aperto no peito ^‡^	Não	591 (32,8)	**<0,001**	625 (34,4)	**<0,001**	646 (35,6)	**<0,001**	264 (26,6)	**<0,001**
	Sim	1213 (67,2)		1191 (65,6)		1168 (64,4)		730 (73,4)	
Diarreia ^‡||^	Não	1235 (68,5)	**<0,001**	1245 (68,6)	**<0,001**	1260 (69,5)	**<0,001**	640 (64,4)	**<0,001**
	Sim	569 (31,5)		571 (31,4)		554 (30,5)		354 (994)	
Rubor facial ^‡^	Não	1427 (79,1)	**<0,001**	1441 (79,4)	**<0,001**	1453 (80,1)	**<0,001**	756 (76,1)	**<0,001**
	Sim	373 (20,7)		371 (20,4)		357 (19,7)		238 (23,9)	
**Características definidoras comportamentais**									
Agitação psicomotora ^‡^	Não	150 (8,3)	**<0,001**	131 (7,2)	**<0,001**	180 (9,9)	**<0,001**	61 (6,1)	**<0,001**
	Sim	1654 (91,7)		1685 (92,8)		1634 (90,1)		933 (93,9)	
Choro fácil ^‡^	Não	490 (27,2)	**<0,001**	483 (26,6)	**<0,001**	521 (28,7)	**<0,001**	214 (21,5)	**<0,001**
	Sim	1314 (72,8)		1333 (73,4)		1293 (71,3)		780 (78,5)	
Impaciência aumentada ^¶^	Não	219 (12,1)	**<0,001**	169 (9,3)	**<0,001**	214 (11,8)	**<0,001**	80 (8,0)	**<0,001**
	Sim	1585 (87,9)		1647 (90,7)		1600 (88,2)		914 (92,0)	
Produtividade diminuída ^‡^	Não	287 (15,9)	**<0,001**	292 (16,1)	**<0,001**	258 (14,2)	**<0,001**	123 (12,4)	**<0,001**
	Sim	1517 (84,1)		1524 (83,9)		1556 (85,8)		871 (87,6)	
Baixa autoestima ^§^	Não	266 (14,9)	**<0,001**	273 (15,1)	**<0,001**	246 (13,7)	**<0,001**	96 (9,7)	**<0,001**
	Sim	1525 (85,1)		1531 (84,9)		1556 (86,3)		898 (90,3)	
**Características definidoras cognitivas**									
Atenção alterada ^‡||^	Não	212 (11,8)	**<0,001**	201 (11,1)	**<0,001**	209 (11,5)	**<0,001**	87 (8,8)	**<0,001**
	Sim	1592 (88,2)		1615 (88,9)		1605 (88,5)		907 (91,2)	
Esquecimento frequente ^‡^	Não	291 (16,1)	**<0,001**	280 (15,4)	**<0,001**	296 (16,3)	**<0,001**	117 (11,8)	**<0,001**
	Sim	1513 (83,9)		1536 (84,6)		1518 (83,7)		877 (88,2)	
**Características definidoras emocionais**									
Preocupação excessiva ^‡§^	Não	110 (6,1)	**<0,001**	96 (5,3)	**<0,001**	126 (6,9)	**<0,001**	24 (2,4)	**<0,001**
	Sim	1694 (93,9)		1720 (94,7)		1688 (93,1)		970 (97,6)	
Frustração ^§^	Não	195 (10,8)	**<0,001**	190 (10,5)	**<0,001**	154 (8,5)	**<0,001**	68 (6,8)	**<0,001**
	Sim	1609 (89,2)		1626 (89,5)		1660 (91,5)		926 (93,2)	
Solidão ^§^	Não	437 (24,2)	**<0,001**	421 (23,2)	**<0,001**	401 (22,1)	**<0,001**	178 (17,9)	**<0,001**
	Sim	1367 (75,8)		1395 (76,8)		1413 (77,9)		816 (82,1)	
Medo ^‡§||^	Não	305 (16,9)	**<0,001**	326 (18,0)	**<0,001**	325 (17,9)	**<0,001**	114 (11,5)	**<0,001**
	Sim	1499 (83,1)		1490 (82,0)		1489 (82,1)		880 (88,5)	
Raiva ^§||¶^	Não	557 (30,9)	**<0,001**	521 (28,7)	**<0,001**	538 (29,7)	**<0,001**	225 (22,6)	**<0,001**
	Sim	1247 (69,1)		1295 (71,3)		1276 (70,3)		769 (77,4)	
Tristeza**	Não	266 (14,7)	**<0,001**	273 (15,0)	**<0,001**	246 (13,6)	**<0,001**	68 (6,8)	**<0,001**
	Sim	1538 (85,3)		1543 (85,0)		1568 (86,4)		926 (93,2)	

*f = Frequência absoluta; ^†^% = Frequência relativa; características definidoras dos diagnósticos de enfermagem; ^‡^Ansiedade; ^§^Síndrome do estresse por mudança; ^||^Síndrome pós-trauma; ^¶^Sobrecarga de estresse; **Tristeza crônica, segundo a taxonomia NANDA-I^(8)^; valor p < 0,05 - estatisticamente significativo

**Tabela 3-:** Características definidoras que se configuram como preditoras de desordens de saúde mental em universitários na era pós-COVID-19 (n = 2.349). Viçosa, MG, Brasil, 2024

		Sintomas de ansiedade		Sintomas de estresse		Sintomas de depressão		Transtorno do estresse pós-traumático	
	Categorias	ORA* (IC 95% ^†^ )	Valor P	ORA* (IC 95% ^†^ )	Valor P	ORA* (IC 95% ^†^ )	Valor P	ORA* (IC 95% ^†^ )	Valor P
**Características definidoras físicas**									
Alteração no ciclo sono-vigília ^§||^	Não ^‡^	-	-	-	-	1		1	
	Sim	-	-	-	-	1,405 (1,054-1,872)	**0,021**	1,437 (1,111-1,858)	**0,006**
Transpiração aumentada ^§^	Não ^‡^	1		1		-	**-**	1	
	Sim	1,463 (1,075-1,991)	**0,015**	1,382 (1,016-1,878)	**0,039**	-	**-**	1,355 (1,108-1,658)	**0,003**
Boca seca ^§^	Não ^‡^	1		-	-	1		1	
	Sim	3,312 (2,369-4,630)	**<0,001**	-	-	1,613 (1,209-2,152)	**0,001**	1,551 (1,265-1,901)	**<0.001**
Padrão respiratório alterado ^§^	Não ^‡^	1		1		-	-	1	
	Sim	1,618 (1,170-2,238)	**0,004**	1,877 (1,353-2,604)	**<0,001**	-	-	1,373 (1,097-1,719)	**0,006**
Palpitações cardíacas ^§¶^	Não ^‡^	1		1		-	-	1	
	Sim	3,316 (2,379-4,623)	**<0,001**	1,455 (1,058-2,002)	**0,021**	-	-	1,319 (1,062-1,638)	**0,012**
Tensão muscular ^§**^	Não ^‡^	-	-	1		-	-	-	-
	Sim	-	-	1,444 (1,072-1,943)	**0,016**	-	-	-	-
Cefaleia ^¶^	Não ^‡^	-	-	1		-	-	1	
	Sim	-	-	1,298 (0,966-1,744)	0,083	-	-	1,299 (1,054-1,599)	**0,014**
Náusea ^§¶^	Nã ^o‡^	1		1		1		1	
	Sim	2,707 (1,822-4,020)	**<0,001**	1,628 (1,109-2,390)	**0,013**	1,952 (1,397-2,726)	**<0,001**	1,226 (0,933-1,514)	0,058
Aperto no peito ^§^	Não ^‡^	1		-	-	-	-	-	-
	Sim	1,422 (1,041-1,941)	**0,027**	-	-	-	-	-	-
**Características definidoras comportamentais**									
Agitação psicomotora ^§^	Não ^‡^	1		1		-	-	1	
	Sim	2,238 (1,614-3,104)	**<0,001**	3,233 (2,312-4,522)	**<0,001**	-	-	1,425 (1,009-2,013)	**0,045**
Choro fácil ^§^	Não ^‡^	1		1		-	-	1	
	Sim	1,550 (1,170-2,053)	**0,002**	1,813 (1,358-2,420)	**<0,001**	-	-	1,343 (1,076-1,678)	**0,009**
Impaciência aumentada ^**^	Não ^‡^	-	-	1		1		-	-
	Sim	-	-	2,225 (1,601-3,093)	**<0,001**	1,319 0,953-1,825)	0,095	-	-
Produtividade diminuída ^§^	Não ^‡^	-	-	-	-	1		-	-
	Sim	-	-	-	-	2,449 (1,827-3,282)	**<0,001**	-	-
Baixa autoestima ^||^	Não ^‡^	1		1		1		1	
	Sim	2,026 (1,476-2,782)	**<0,001**	1,632 (1,189-2,239)	**0,002**	2,205 (1,649-2,949)	**<0,001**	1,696 (1,272-2,260)	**<0,001**
**Características definidoras cognitivas**									
Atenção alterada ^§¶^	Não ^‡^	-	-	1		-	-	-	-
	Sim	-	-	1,649 (1,169-2,325)	**0,004**	-	-	-	-
Esquecimento frequente ^§^	Não ^‡^	1		1		1		1	
	Sim	1,433 (1,061-1,934)	**0,019**	1,644 (1,197-2,257)	**0,002**	1,362 (1,009-1,838)	**0,043**	1,547 (1,189-2,013)	**0,001**
**Características definidoras emocionais**									
Preocupação excessiva ^§||^	Não ^‡^	1		1		1		1	
	Sim	1,728 (1,209-2,471)	**0,003**	2,698 (1,868-3,897)	**<0,001**	1,539 (1,075-2,205)	**0,019**	2,825 (1,764-4,523)	**<0,001**
Frustração ^||^	Não ^‡^	1		-	-	1		-	-
	Sim	1,412 (0,991-2,012)	0,056	-	-	2,454 (1,793-3,359)	**<0,001**	-	-
Solidão ^||^	Não ^‡^	-	-	1		1		1	
	Sim	-	-	1,677 (1,237-2,273)	**0,001**	1,822 (1,378-2,407)	**<0,001**	1,420 (1,120-1,801)	**0,004**
Medo ^§||¶^	Não ^‡^	1		-	-	1		1	
	Sim	2,036 (1,508-2,748)	**<0,001**	-	-	1,305 (0,972-1,152)	**0,076**	1,677 (1,281-2,196)	**<0,001**
Raiva ^||¶**^	Não ^‡^	-	-	1		1		1	
	Sim	-	-	2,213 (1,641-2,986)	**<0,001**	1,923 (1,446-2,557)	**<0,001**	1,597 (1,287-1,983)	**<0,001**
Tristeza ^††^	Não ^‡^	1		1		1		1	
	Sim	1,487 (1,062-2,082)	**0,021**	1,572 (1,118-2,211)	**0,009**	2,557 (1,871-3,472)	**<0,001**	1,522 (1,091-2,123)	**0,013**

*ORA = *Odds Ratio* Ajustado; ^†^IC 95% = Intervalo de confiança a 95%; ^‡^Referência para o ORA, características definidoras dos diagnósticos de enfermagem; ^§^Ansiedade; ^||^Síndrome do estresse por mudança; ^¶^Síndrome pós-trauma; ^**^Sobrecarga de estresse; ^††^Tristeza crônica, segundo a taxonomia NANDA-I^(8)^; valor p < 0,05 - estatisticamente significativo

## Discussão

Este estudo determinou características definidoras físicas, comportamentais, cognitivas e emocionais dos diagnósticos de enfermagem, da taxonomia da NANDA-I^(8)^, Ansiedade (00146); Sobrecarga de Estresse (00177); Síndrome Pós-trauma (00141); Síndrome do Estresse por Mudança (00114) e Tristeza Crônica (00137) que se configuraram como preditoras naqueles estudantes universitários com desordens de saúde mental na era pós-COVID-19. Tanto as características definidoras de diagnósticos de enfermagem observados quanto as desordens de saúde mental foram altamente prevalentes nessa população, o que torna esses achados preocupantes.

Os problemas de saúde mental foram fortemente agravados na pandemia da COVID-19 e perpetuaram após o seu término, inclusive em estudantes de graduação. Investigações realizadas com esse público já relataram a prevalência desses problemas ao redor do mundo, na era pós-COVID-19^(6-7,9)^. Contudo, encontramos maiores prevalências dessas desordens de saúde mental nos estudantes brasileiros. Isso denota o grande sofrimento mental vivenciado na era pós-pandemia, e a necessidade urgente de estabelecimento de políticas de cuidado que prezem pela saúde mental dos mesmos.

Em relação às características definidoras físicas, transpiração aumentada, padrão respiratório alterado e palpitações cardíacas emergiram como preditoras para ansiedade, estresse e TEPT; boca seca para ansiedade, TEPT e depressão; náusea para ansiedade, estresse e depressão; aperto no peito para ansiedade e depressão; alteração no ciclo sono-vigília para TEPT e depressão; tensão muscular para estresse; e cefaleia para TEPT.

Segundo a NANDA-I^(8)^, todos esses sinais e sintomas físicos, à exceção da cefaleia, são características definidoras do diagnóstico Ansiedade. O diagnóstico Síndrome do Estresse por Mudança apresenta também, como características definidoras, a referência a sintomas físicos aumentados. Já o diagnóstico Sobrecarga de Estresse, refere-se ao modo de expressar tensão. Palpitações cardíacas e cefaleia constam no diagnóstico Síndrome Pós-trauma. Enquanto isso, o diagnóstico Tristeza Crônica não apresenta características definidoras físicas. Esses achados reafirmam que as características definidoras de fato estão presentes em pessoas com ansiedade, estresse e depressão; e, além disso, podem levar ao desenvolvimento de tais condições clínicas, já que se configuraram como variáveis preditoras no presente estudo.

No contexto da pandemia da COVID-19, alterações no padrão de sono foram frequentes nos estudantes universitários^(17)^. No presente estudo, essa prevalência também foi expressiva para as quatro desordens investigadas (maior que 80,0%), demonstrando que as alterações no ciclo sono-vigília se mantiveram na era pós-COVID-19. Esses dados são preocupantes, pois a alteração no padrão de sono tem correlação direta com problemas de saúde mental em universitários^(12)^.

Manifestações como transpiração aumentada, boca seca, parâmetros cardíacos e respiratórios alterados e alterações gastrointestinais, como a náusea, também podem estar presentes em pessoas com ansiedade^(8)^. Essas manifestações também são investigadas em instrumentos validados para avaliar ansiedade, estresse, depressão, como a DASS-21^(14)^, e na IES-R para avaliação do TEPT^(15)^. Isso reafirma a importância da avaliação dessas características.

No presente estudo, as prevalências de boca seca, palpitações cardíacas e padrão respiratório alterado foram expressivas nos estudantes que manifestaram ansiedade (54,3%, 64,3%, 64,7%, respectivamente). E, principalmente os parâmetros cardíacos e respiratórios também foram expressivos nos estudantes que apresentaram TEPT (69% e 71,4% respectivamente) e estresse (61,8% e 63,8% respectivamente). A ativação do eixo hipotálamo-hipófise-adrenal e do sistema nervoso simpático em situações de estresse justificam essas manifestações^(18)^. No contexto pós-pandêmico, as prevalências expressivas de TEPT e de estresse nos estudantes revelam a importância de avaliar essas manifestações nesse público.

A tensão muscular emergiu como característica definidora preditora para a presença de estresse, com prevalência expressiva nos estudantes do presente estudo (80,1%). Como o sistema nervoso autônomo inerva praticamente todos os sistemas orgânicos do corpo, a ativação da resposta simpática pode levar a várias alterações fisiológicas, como, por exemplo, o aumento da tensão e da dor muscular^(19)^. Em estudantes universitários, no contexto pandêmico, pesquisadores observaram alta prevalência de tensão nos músculos da face e os níveis de estresse foram significativamente maiores^(20)^. Da mesma forma, a cefaleia também pode ocorrer e estar associada aos elevados níveis de estresse em estudantes^(21)^. De fato, no presente estudo, esta característica definidora foi preditora para o desenvolvimento do TEPT, com alta prevalência nos estudantes (70,2%).

Outro sintoma físico que configurou como característica definidora preditora de ansiedade e de depressão foi a sensação de aperto no peito, com prevalência de 67,2% em estudantes com ansiedade e 64,4% com depressão. Estudo realizado com estudantes universitários encontrou associação significativa entre a presença de ansiedade e o sintoma aperto no peito ou no coração; o mesmo não ocorreu para a depressão^(22)^. É importante destacar que o sintoma aparece como característica definidora do diagnóstico Ansiedade, na NANDA-I^(8)^. Mas, devido à sua alta prevalência em estudantes com sintomas de depressão e ao fato dele ter emergido como variável preditora para essa condição, sugere-se avaliar a possibilidade de inclusão desse sintoma como característica definidora do diagnóstico Tristeza Crônica.

Ao considerar que as características definidoras físicas são mais facilmente perceptíveis, elas podem atuar como sinais de alerta para tais condições. No contexto universitário, por exemplo, quando os professores identificarem a presença das referidas manifestações nos estudantes em sala de aula, uma maior atenção deve ser dada sobretudo em relação à sua saúde mental, para que os problemas emocionais sejam identificados em estágios iniciais e intervenções precoces possam ser implementadas.

Dentre as características definidoras comportamentais identificadas nos estudantes, baixa autoestima foi preditora para ansiedade, estresse, depressão e TEPT; agitação psicomotora e o choro fácil para ansiedade, estresse e TEPT; impaciência aumentada para estresse; e produtividade diminuída para depressão. Segundo a NANDA-I^(8)^, a baixa autoestima é característica definidora do diagnóstico Síndrome do Estresse por Mudança; agitação psicomotora, choro fácil e produtividade diminuída são características do diagnóstico Ansiedade; e impaciência aumentada configura-se como característica definidora de Sobrecarga de Estresse.

A baixa autoestima pode ser entendida como a percepção negativa sobre o valor próprio, a autoaceitação, o autorrespeito, a competência e a atitude em relação a si mesmo^(8)^. No contexto universitário, a baixa autoestima é um fator de influência para níveis mais elevados de depressão^(23)^, além de comprometer significativamente o desempenho acadêmico^(24)^. Tal fato foi agravado com a pandemia e perpassa pelo contexto pós-pandêmico, ao observarmos a expressiva prevalência de baixa autoestima, que girou em torno de 84,9% a 90,1% nos estudantes com desordens de saúde mental desse estudo. Diante disso, a avaliação da autoestima nos estudantes pode contribuir para o estabelecimento dos diagnósticos relacionados à saúde mental de forma mais acurada e, consequentemente, no estabelecimento de intervenções mais efetivas.

Agitação psicomotora e choro fácil também foram altamente prevalentes (aproximadamente 92,0% e 75,0%, respectivamente) nos estudantes com ansiedade, estresse e TEPT. A DASS-21^(14)^ apresenta, no construto ansiedade, a presença de tremores, por exemplo nas mãos e, no construto estresse, dificuldades de se acalmar e de relaxar, e agitação. Esses sintomas estão relacionados à agitação psicomotora, o que reforça a importância da sua avaliação em pessoas com sintomas de ansiedade, estresse e em situações pós-traumáticas. Já o choro fácil, que emergiu como preditor para essas condições, também é apresentado pela DASS-21^(14)^, mas de forma indireta como “senti que estava um pouco emotivo/sensível demais” no construto depressão. Reafirma-se a necessidade de detectar essas características definidoras nos estudantes, que foram fortemente impactados a nível psicológico, pela pandemia da COVID-19.

A impaciência aumentada também foi expressiva em estudantes que apresentaram sintomas de estresse (90,7%) no contexto pós-pandemia. Este também é um sintoma avaliado pela IES-R em pessoas com TEPT (“eu me sentia irritável e bravo”)^(15)^. As diversas consequências da pandemia na forma de ensino levaram os estudantes a desenvolverem sentimentos de impaciência, aborrecimento e hostilidade^(25)^, e isso pode predispor ao desenvolvimento de estresse, como foi evidenciado pelo presente estudo. Visto isso, torna-se importante trabalhar com estratégias de enfrentamento e alívio de estresse com essa população, de forma que esses sentimentos negativos não impactem na performance acadêmica do estudante.

Outro sintoma frequente nos estudantes deste estudo e que se configurou como preditor para a depressão foi a produtividade diminuída (85,8%). Destaca-se que a falta de vontade em realizar as atividades do dia a dia é uma característica da depressão. No contexto universitário, essa falta de vontade pode refletir na diminuição da produtividade, com consequente impacto no desempenho acadêmico. Contudo, na NANDA-I, o diagnóstico Tristeza Crônica não apresenta essa característica como definidora^(8)^. Devido à alta prevalência evidenciada por este estudo e sua relação direta com a depressão, sugere-se a possibilidade de inclusão.

Dentre os sinais e sintomas cognitivos, esquecimento frequente foi preditor para ansiedade, estresse, TEPT e depressão; e atenção alterada para estresse e TEPT. Segundo a NANDA-I, o esquecimento configura-se como característica definidora do diagnóstico Ansiedade; e a atenção alterada como característica definidora de Ansiedade e Síndrome Pós- trauma. Ainda, a IES-R apresenta os problemas de concentração como características do TEPT^(15)^, reforçando a necessidade de avaliá-los.

Durante a pandemia da COVID-19, a dificuldade de concentração foi frequente entre os estudantes universitários, com prevalência em torno de 89%^(17)^. Os problemas cognitivos perduraram até a era pós-pandemia, fato comprovado pelo presente estudo, que encontrou prevalência de esquecimento frequente maior que 80% em todos os construtos avaliados, e de atenção alterada de 88,9% e 91,2% em estudantes com estresse e TEPT, respectivamente. Os achados são importantes, pois a presença das características definidoras pode comprometer o desempenho acadêmico dos estudantes, reduzir a motivação para os estudos e, em última instância, contribuir para a evasão escolar.

Dentre as características definidoras emocionais, preocupação excessiva e tristeza foram preditoras para ansiedade, estresse, TEPT e depressão; solidão e raiva para estresse, TEPT e depressão; medo para ansiedade e TEPT; e frustração para depressão.

Observou-se, no presente estudo, alta prevalência de sentimentos de preocupação (>93,0% em todos os construtos) e tristeza (≥85,0% em todos os construtos) nos estudantes. Em estudo realizado com 195 estudantes universitários para compreender os efeitos da pandemia na sua saúde mental, encontrou-se que a preocupação com o desempenho acadêmico e os pensamentos excessivos e depressivos também estavam presentes nesta população; além disso, tais pensamentos contribuíram para a ocorrência de pensamentos suicidas^(17)^. Diante dos achados, fica evidente como esses sentimentos ainda permanecem no contexto pós-pandêmico, e que, quando exacerbados, podem comprometer a saúde mental dos estudantes e leva-los a comportamentos de risco. Portanto, ao avaliar a saúde mental dos estudantes, sugere-se observar a presença desses sentimentos ao elencar todos os diagnósticos de enfermagem aqui estudados.

Sentimentos de solidão^(17)^ e raiva^(26)^, que se fizeram presente em estudantes durante a pandemia, também se mostraram altamente prevalentes na era pós-COVID-19. De fato, mais de 75,0% e de 69,0% dos estudantes deste estudo apresentaram esses sintomas, respectivamente. Segundo a NANDA-I, esses sentimentos estão presentes nos diagnósticos de Síndrome do Estresse por Mudança, Síndrome Pós-trauma e Sobrecarga de Estresse^(8)^. Contudo, no presente estudo, eles também emergiram como preditores para depressão em estudantes de graduação, de forma que sugerimos a avaliação desses sentimentos também em quadros de tristeza.

O medo, que também foi relatado por estudantes universitários no contexto pandêmico^(17)^, fez-se presente na era pós-COVID-19 de forma muito frequente, conforme evidenciado pelo nosso estudo, principalmente nos estudantes com ansiedade (83,1%) e com TEPT (88,5%). O medo pode influenciar múltiplos aspectos da vida e da saúde das pessoas^(27)^. Durante a pandemia, a reação desencadeou sérias consequências fisiopatológicas, sociais, comportamentais e mentais^(27)^. De fato, o estudo apontou que a maior prevalência de medo da COVID-19 foi relacionada a pior qualidade do sono, pior percepção de saúde, tristeza, maior estresse, sintomas depressivos e ideação suicida^(27)^. Assim, compreender e mitigar o medo na população de estudantes universitários é uma grande preocupação e um ponto focal para intervenções^(28)^. Destaca-se ainda, que segundo a NANDA-I^(8)^, esse sentimento está presente nos diagnósticos Ansiedade, Síndrome do Estresse por Mudança e Síndrome Pós-trauma, demonstrando que os resultados encontrados estão de acordo com a taxonomia.

Por fim, frustração foi outro sintoma expressivo no presente estudo, com prevalência de 91,5% em estudantes com depressão. Durante a pandemia, os estudantes expressaram frustrações em relação às aulas remota; ao acesso às tecnologias; à interrupção de atividades de pesquisa e extensão; e aos aspectos familiares, sociais, emocionais, comportamentais e financeiros da vida^(29)^. E, com os achados aqui apresentados, nota-se que esse sentimento ainda permanece na era pós-COVID-19. Garantir, então, que as frustrações dos estudantes e as barreiras ao sucesso sejam reconhecidas e consideradas pode ajudar a evitar abandono ao ensino superior^(29)^.

Diante dos resultados desta investigação, sugerimos que as características definidoras físicas, comportamentais, cognitivas e emocionais que emergiram como preditores de ansiedade, estresse, TEPT e depressão nos estudantes de graduação na era pós-COVID-19 sejam melhor estudados como possíveis características definidoras dos respectivos diagnósticos de Ansiedade; Síndrome do estresse por mudança; Síndrome pós-trauma; Sobrecarga de estresse; Tristeza crônica. Elas podem proporcionar mais pistas diagnósticas e, consequentemente, facilitar a implementação de intervenções efetivas para o público.

Este estudo apresenta limitações que devem ser destacadas. Em primeiro lugar, a coleta de dados de forma *online* impediu o cálculo de uma taxa precisa de respostas; além disso, estudantes sem acesso à internet não conseguiram participar. Em pesquisas futuras, a coleta de dados presencial evitaria a ocorrência do viés de seleção. Segundo, foram utilizados questionários de autorrelato para avaliar sintomas relacionados às desordens de saúde mental, bem como a presença das características definidoras dos diagnósticos de enfermagem estudados. Para estudos futuros, sugere-se a realização de diagnósticos clínicos por médicos e a avaliação das características definidoras por enfermeiros, de forma que esses dados não sejam baseados apenas na observação do participante. Um terceiro aspecto a ser mencionado se refere ao fato de o estudo ter sido limitado a apenas uma região do país, o que pode restringir, em partes, a generalização dos resultados para outras regiões ou países. Contudo, o expressivo tamanho amostral, proveniente de 15 *campi* (em diferentes cidades) e 94 cursos de graduação aumentou a confiabilidade e validade das comparações feitas. Além disso, as associações com ORA inferior a 2,0 devem ser interpretadas com cautela, uma vez que podem produzir uma associação falso positiva^(30)^. Sugere-se, ainda, considerar na análise estatística o semestre acadêmico, uma vez que estudantes de diferentes semestres podem apresentar características diferentes. Por fim, ressaltamos que não foi utilizada a versão mais atual da NANDA-I, pois, no momento da coleta de dados, ela ainda não havia sido lançada. Contudo, observou-se pelos pesquisadores a não contemplação das características definidoras investigadas neste estudo.

Apesar das limitações, este estudo traz avanços ao conhecimento científico, pois está entre os primeiros a identificar a prevalência de desordens relacionadas à saúde mental entre estudantes de graduação na era pós-COVID-19 no Brasil, e em especial para área da enfermagem, uma vez que evidenciou características definidoras de diagnósticos de enfermagem que se configuraram como preditoras de ansiedade, estresse, depressão e TEPT. Os achados podem auxiliar enfermeiros a aprimorarem a avaliação das desordens de saúde mental na população de estudantes universitários, bem como para a implementação de intervenções eficazes para promoção da saúde mental.

Além disso, ressalta-se que a versão mais atual da NANDA-I, lançada em abril de 2024^(31)^, não contempla os diagnósticos Síndrome do Estresse por Mudança, Sobrecarga de Estresse e Tristeza Crônica. Ao considerar a alta prevalência das características definidoras desses diagnósticos em uma amostra expressiva de estudantes de graduação e que foram preditoras para ansiedade, estresse, depressão e TEPT, este estudo valida a ocorrência das mesmas. Dessa forma, sugere-se a reinserção dos diagnósticos, com suas respectivas características definidoras, na taxonomia da NANDA-I.

## Conclusão

Este estudo evidenciou características definidoras físicas, comportamentais, cognitivas e emocionais presentes nos diagnósticos de enfermagem Ansiedade; Síndrome do Estresse por Mudança; Síndrome Pós-trauma; Sobrecarga de Estresse; Tristeza Crônica, segundo a NANDA-I, que se configuraram como preditoras para o desenvolvimento de desordens de saúde mental em estudantes universitários na era pós-COVID-19, tais como: alteração no ciclo sono-vigília; transpiração aumentada; boca seca; padrão respiratório alterado; palpitações cardíacas; tensão muscular; cefaleia; náusea; aperto no peito; agitação psicomotora; choro fácil; impaciência aumentada; produtividade diminuída; baixa autoestima; atenção alterada; esquecimento frequente; preocupação excessiva; frustração; solidão; medo; raiva; e tristeza.

## Data Availability

O conjunto de dados deste artigo está disponível na página da RLAE no repositório SciELO Data, no link https://doi.org/10.48331/scielodata.D4GINI

# Translation: Características definitorias predictoras de trastornos de salud mental entre estudiantes de pregrado en la era post COVID-19: estudio transversal multicéntrico*

**DOI:** 10.1590/1518-8345.7741.4670

**Authors:** Caroline de Castro Moura, Luana Vieira Toledo, Gabriela Tavares Boscarol, Rafael Lopes Chaves, Denismar Alves Nogueira, Carine Alves da Costa Marinho, Bruna de Oliveira Alves, Bárbara Guimarães Lourenço, Érika de Cássia Lopes Chaves, Tânia Couto Machado Chianca

**Affiliations:** 1 Universidade Federal de Viçosa, Departamento de Medicina e Enfermagem, Viçosa, MG, Brasil.; 2 Universidade Federal de Minas Gerais, Escola de Enfermagem, Belo Horizonte, MG, Brasil.; 3 Becaria de la Coordenação de Aperfeiçoamento de Pessoal de Nível Superior (CAPES), Brasil.; 4 Universidade Professor Edson Antônio Velano, Curso de Psicologia, Alfenas, MG, Brasil.; 5 Becaria (o) de la Fundação de Amparo à Pesquisa do Estado de Minas Gerais (FAPEMIG), Brasil.; 6 Universidade Federal de Alfenas, Departamento de Estatística, Alfenas, MG, Brasil.; 7 Universidade Federal de Alfenas, Escola de Enfermagem, Alfenas, MG, Brasil.

**Keywords:** Signos y Síntomas, Salud Mental, Universidades, Estudiantes, COVID-19, Enfermería

## Abstract

evaluar características definitorias de diagnósticos de enfermería que se configuran como predictoras de trastornos de salud mental entre estudiantes de pregrado en la era post COVID-19.

estudio transversal, multicéntrico, realizado con estudiantes de pregrado de cuatro universidades públicas del estado de Minas Gerais. Los datos fueron recolectados mediante un cuestionario estructurado, en línea, con variables sociodemográficas y clínicas. Se investigó, además, mediante autoinforme, la presencia de características definitorias incluidas en los Diagnósticos de Enfermería de Ansiedad, Síndrome del Estrés por Cambio; Síndrome Postraumático; Sobrecarga de Estrés y Tristeza Crónica, presentes en la taxonomía de la NANDA-I. Se utilizaron también la *Depression*, *Anxiety and Stress Scale* y la *Impact of Events Scale-Revised*. Se realizó un análisis de regresión logística multivariada para determinar las características definitorias que se configuraron como predictoras de los trastornos de salud mental. Se estimaron *odds ratios* ajustadas, junto con los intervalos de confianza del 95%, con un nivel de significancia del 5%.

participaron en el estudio 2.349 estudiantes de pregrado. Se investigó la presencia de 24 características definitorias físicas, conductuales, cognitivas y emocionales de los diagnósticos de enfermería en cuestión. De estas, 13 emergieron como predictoras para los síntomas de ansiedad; 15 para los síntomas de estrés; 11 para los síntomas de depresión; y 15 para el trastorno de estrés postraumático.

se evidenciaron características definitorias físicas, conductuales, cognitivas y emocionales de diagnósticos de enfermería de la NANDA-I que se configuraron como predictoras para el desarrollo de trastornos de salud mental en universitarios en la era post COVID-19.

## Destacados

**(1)** Los trastornos de salud mental son prevalentes en universitarios en la era post COVID-19. **(2)** Los diagnósticos de enfermería relacionados con la salud mental son prevalentes en estudiantes. **(3)** Características definitorias emergieron como predictoras de trastornos de salud mental. **(4)** Es necesario implementar intervenciones para promover la salud mental en esta población

## Introducción

Según la *World Health Organization*, la salud mental se define como “un estado de bienestar en el cual el individuo es consciente de sus propias capacidades, puede afrontar las tensiones normales de la vida, trabajar de forma productiva y contribuir a su comunidad” ^( 1 )^. Los trastornos relacionados con ella representan un gran problema de salud pública a nivel mundial y surgen cuando la persona no logra adaptarse a los cambios que ocurren en su vida, generando un gran sufrimiento ^( 1 )^.

En todo el mundo, una de cada ocho personas vive con trastornos de salud mental ^( 1 )^. Los resultados del *Global Burden of Diseases* señalaron que entre los años 1990 y 2019, el número global de años de vida ajustados por discapacidad debido a trastornos mentales aumentó de 80,8 millones a 125,3 millones, con una gran carga para las personas en todo el mundo ^( 2 )^.

Los estudiantes de pregrado representan una población vulnerable al desarrollo de trastornos de salud mental ^( 3 )^, y la pandemia de COVID-19 los agravó aún más ^( 4 - 5 )^, pues, entre sus numerosos impactos negativos, desencadenó una crisis global en la salud mental de las personas ^( 1 )^. De hecho, la ansiedad, el estrés, la depresión y el trastorno de estrés postraumático (TEPT) continuaron siendo prevalentes en la era post COVID-19 ^( 6 - 7 )^.

En el contexto de la enfermería, la taxonomía de la NANDA-I ^( 8 )^ presenta algunos diagnósticos relacionados con la salud mental que pueden estar presentes en la población universitaria, y que probablemente fueron intensificados debido al contexto pandémico. Entre ellos, se destacan la Ansiedad (00146); Sobrecarga de Estrés (00177); Síndrome Postraumático (00141); Síndrome del Estrés por Cambio (00114) y Tristeza Crónica (00137). Fijarse a las características definitorias y a los factores relacionados que pueden desencadenar estos problemas, con el fin de implementar medidas efectivas para solucionarlos y prevenirlos, resulta esencial en este contexto de crisis de salud mental.

A nivel global, ya se han evaluado trastornos de salud mental en estudiantes universitarios en la era post COVID-19 ^( 6 - 7 , 9 - 12 )^. Por ejemplo, un estudio realizado en China ^( 6 )^ identificó que factores asociados a cambios sociales, familiares y económicos impuestos por la pandemia aumentaron el riesgo de síntomas psicológicos en estudiantes universitarios. Otro estudio ^( 7 )^, realizado en Chile, identificó, además de altas prevalencias de depresión, ansiedad y estrés, que el sexo femenino, pertenecer a minorías sexuales y el uso de psicofármacos parecen tener impacto en la susceptibilidad a problemas de salud mental. Y un estudio realizado en Tailandia ^( 12 )^ encontró que condiciones médicas, malas relaciones con familiares, amigos u otras personas, tener problemas mientras se cursaban estudios universitarios y el impacto autopercibido de la COVID-19 en la vida del estudiante estuvieron todos asociados a problemas de salud mental.

Cabe destacar que gran parte de los estudios involucraron, principalmente, el rastreo del estado psicológico y su asociación con factores demográficos, clínicos y académicos ^( 6 - 7 , 9 - 12 )^. No obstante, las investigaciones en Brasil son escasas y, hasta el momento, no se encontraron, a nivel nacional o internacional, estudios de revisión sistemática o de alcance, ni estudios que evaluaran la presencia de características definitorias de diagnósticos de enfermería en estudiantes de pregrado en la era post COVID-19 y las estimaran como predictoras de ansiedad, estrés, depresión y TEPT. Evaluar estas características en una población vulnerable al desarrollo de trastornos de salud mental y que vivió una gran crisis sanitaria puede ser un paso importante en el desarrollo de estrategias institucionales de cuidado para este público. En este sentido, el presente estudio tuvo como objetivo evaluar características definitorias de diagnósticos de enfermería que se configuran como predictoras de trastornos de salud mental entre estudiantes de pregrado en la era post COVID-19.

## Método

### Diseño del estudio

Estudio transversal, multicéntrico, realizado con estudiantes universitarios de pregrado y reportado de acuerdo con las recomendaciones del *Strengthening the Reporting of Observational Studies in Epidemiology* ^( 13 )^.

### Lugar y período

El estudio se llevó a cabo en cuatro universidades públicas del estado de Minas Gerais, Brasil, entre enero de 2023 y abril de 2024. La universidad 1 cuenta con 79 cursos de pregrado distribuidos en cinco campus en las regiones Metropolitana y Norte del estado de Minas Gerais; la universidad 2 ofrece 56 cursos de pregrado en tres campus en la región Metropolitana; la universidad 3 dispone de 67 cursos de pregrado en tres campus en la *Zona da Mata*, región Metropolitana y *Alto Paranaíba*/Triángulo Mineiro; por último, la universidad 4 está compuesta por 38 cursos de pregrado en cuatro campus ubicados en el Sur y Suroeste del estado.

### Población y definición de la muestra

La población estuvo compuesta por 67.110 estudiantes universitarios de pregrado, siendo 33.900 en la universidad 1, 13.110 en la universidad 2, 14.000 en la universidad 3 y 6.100 en la universidad 4. Para el cálculo de la muestra se utilizó una estimación conservadora de prevalencia del 50%, ya que la prevalencia de trastornos de salud mental en la era post COVID-19 en Brasil era desconocida en el momento de la planificación del estudio. Se utilizó la fórmula n = (z².p(1-p)/e²)/1+(z².p(1-p)/e²N), donde: z = valor z (para un nivel de confianza del 95%, z = 1,96); p = prevalencia de casos con la característica estudiada; e = margen de error; N = tamaño de la población. Al considerar la población de 67.110 estudiantes y un margen de error del 2%, el tamaño mínimo estimado de la muestra para el presente estudio fue de 2.319 estudiantes.

### Criterios de selección

Se reclutaron estudiantes de 18 años o más, que estuvieran regularmente matriculados en cualquier periodo de los cursos de pregrado y que tuvieran disponibilidad de horario para responder a los instrumentos de recolección de datos. Se excluyeron del estudio aquellos que no tenían acceso a internet en el momento de la recolección de los datos y quienes no completaron todo el cuestionario.

### Variables del estudio

La presencia o ausencia de síntomas de ansiedad, estrés, depresión y TEPT fueron consideradas variables dependientes. Las características definitorias de los diagnósticos de enfermería estudiados fueron consideradas explicativas o predictoras. Las características sociodemográficas y clínicas fueron consideradas covariables.

### Instrumentos utilizados para la recolección de la información

Se recopilaron las siguientes características sociodemográficas y clínicas mediante un cuestionario estructurado en línea: sexo biológico; color de piel; estado civil; tipo de vivienda; ingreso familiar (en salarios mínimos de Brasil – en el momento de la recolección de datos: R$1.320,00); actividad laboral; asistencia estudiantil; beca estudiantil (de docencia, investigación o extensión); diagnóstico clínico de trastornos de salud mental; uso de psicofármacos; seguimiento con psiquiatra y psicólogo. Además, se investigó, por medio de autoinforme, la presencia de características definitorias de los siguientes diagnósticos de enfermería, según la taxonomía NANDA-I ^( 8 )^:

1) Ansiedad: agitación psicomotora; llanto fácil; inseguridad; alteración en el ciclo sueño-vigilia; productividad disminuida; opresión en el pecho; boca seca; diarrea; tensión muscular; palpitaciones; náuseas; patrón respiratorio alterado; rubor facial; sudoración aumentada; atención alterada; olvidos; preocupación; miedo.
2) Síndrome del estrés por cambio: baja autoestima; rabia; miedo; preocupación; frustración; alteración en el ciclo vigilia-sueño; soledad.
3) Sobrecarga de estrés: rabia; impaciencia aumentada; tensión muscular.
4) Síndrome postraumático: atención alterada; cefalea; rabia; miedo; palpitaciones.
5) Tristeza crónica: tristeza.

Estas características definitorias fueron seleccionadas por haber sido observadas con mayor frecuencia en estudiantes de pregrado, según la práctica clínica de seis investigadores del equipo, que son enfermeros y docentes universitarios. Además, algunas de estas características también están presentes en la *Depression*, *Anxiety*, *and Stress Scale* (DASS-21) ^( 14 )^ y en la *Impact of Events Scale-Revised* (IES-R) ^( 15 )^.

Para medir los síntomas de ansiedad, estrés y depresión se utilizó la DASS-21 ^( 14 )^. La escala cuenta con 21 ítems en formato de autoinforme, divididos en tres subescalas que evalúan los síntomas percibidos durante la última semana, mediante siete preguntas con cuatro opciones de respuesta (0 = no se aplicó en absoluto a 3 = se aplicó mucho o la mayor parte del tiempo). La suma de cada subescala, multiplicada por dos, proporciona la puntuación total de cada constructo ^( 14 )^. La escala fue traducida y validada para la versión brasileña y presenta adecuadas propiedades psicométricas ^( 14 )^. En la muestra actual, el alfa de Cronbach global fue de 0.944 (para las subescalas de ansiedad, estrés y depresión fue de 0.867, 0.886 y 0.901, respectivamente) ^( 16 )^.

Para evaluar el TEPT se utilizó la IES-R ^( 15 )^. La IES-R es autoaplicable y presenta 22 ítems subdivididos en tres subescalas de síntomas: evitación, intrusión e hiperestimulación, que consideran los últimos siete días. Cada ítem incluye cinco opciones de respuesta (0 - nada en absoluto a 4 - extremadamente). El resultado general es la suma de los promedios de cada subescala. Un valor superior a 5,6 indica la presencia de TEPT ^( 15 )^. El instrumento fue validado y adaptado a la versión brasileña ^( 15 )^, y en la muestra presente, el alfa de Cronbach fue de 0.961 ^( 16 )^.

### Recolección de datos

El estudio se condujo en línea, mediante la plataforma *Google Forms*, durante tres semestres, con el fin de alcanzar el número de participantes estimado por el cálculo muestral. El estudio fue divulgado en los canales oficiales de comunicación de las universidades, en los correos electrónicos institucionales y por medio de la entrega de folletos. Se proporcionó a todos los posibles participantes información detallada sobre los objetivos de la investigación para que pudieran tomar una decisión informada sobre su participación. Aquellos que aceptaron, firmaron el consentimiento informado en formato digital. A continuación, respondieron por autoinforme a los instrumentos de recolección de datos. Los datos fueron almacenados en formato electrónico protegido con contraseña.

### Tratamiento y análisis de los datos

A partir de las respuestas obtenidas por medio del *Google Forms*, se generó una base de datos en el *software Microsoft Office Excel*, versión 2021. Los datos fueron importados al *software IBM SPSS Statistics for Windows*, versión 26 (IBM Corp., Armonk, NY, USA) para su análisis. Las variables estudiadas se resumieron mediante frecuencias absolutas y relativas, media y desviación estándar. Se utilizó el análisis bivariado mediante la prueba de Chi-cuadrado de Pearson para evaluar la asociación entre las características definitorias de los diagnósticos de enfermería estudiados y los síntomas de ansiedad, estrés, depresión y TEPT evaluados con las escalas utilizadas.

Para el análisis de regresión múltiple, también se evaluó la multicolinealidad de las variables. La prueba VIF para colinealidad presentó valores cercanos a 1, sin indicar problemas de multicolinealidad. La presencia o ausencia de síntomas de ansiedad, estrés, depresión y TEPT fueron consideradas variables dependientes. Las variables explicativas (características definitorias de los diagnósticos de enfermería estudiados) se incluyeron en el modelo de regresión logística múltiple mediante el método Backward. Se estimaron los *odds ratios* ajustados (ORA), junto con sus intervalos de confianza del 95% (IC 95), que se utilizaron para determinar aquellas características definitorias que podrían configurarse como predictoras de los síntomas de ansiedad, estrés, depresión y TEPT. La asociación de las variables se consideró estadísticamente significativa cuando p<0,05.

Las variables sociodemográficas y clínicas se incluyeron en el grupo de variables del modelo de regresión logística múltiple; sin embargo, como no fueron estadísticamente significativas (p>0,05), se optó por mantener únicamente las características definitorias de los diagnósticos de enfermería en cuestión.

## Aspectos éticos

Se siguieron rigurosamente las normas éticas para investigaciones que involucran a seres humanos. El estudio fue aprobado por el Comité de Ética en Investigación bajo los pareceres número 5.700.107/ CAAE: 63318222.0.0000.5153. Todos los estudiantes que aceptaron participar en el estudio firmaron el Consentimiento Informado.

## Resultados

Un total de 2.409 estudiantes de pregrado fueron evaluados, de los cuales el 97,5% (2.349) completaron el estudio. El 2,5% (60) fueron excluidos del análisis, ya que eran estudiantes de posgrado (48); rechazaron participar en el estudio (7); no respondieron completamente el cuestionario (3); o no tenían 18 años (2).

La edad de los participantes varió entre 18 y 62 años, con una media de 23,4 años (desviación estándar: 5,4). El 48,6% (1.141) estaban matriculados en cursos del área de ciencias biológicas y de la salud; el 24,2% (569) en ciencias humanas, artes y letras; el 21,3% (501) en ciencias exactas y tecnológicas; y el 5,9% (138) en ciencias agrarias. La descripción de las características sociodemográficas y clínicas se encuentra en la Tabla 1.

**Tabla 1-:** Características sociodemográficas y clínicas de los estudiantes universitarios de pregrado (n = 2.349). Viçosa, MG, Brasil, 2024

**Variables**	f*	**(%** ^†^ **)**
**Sexo biológico**		
Masculino	572	24,4
Femenino	1.777	75,6
**Color de piel**		
Blanco	1.353	57,6
Amarillo	27	1,1
Pardo	676	28,8
Negro	287	12,2
Indígena	6	0,3
**Estado civil**		
Soltero	2.203	93,8
Casado/unión estable	132	5,6
Viudo	1	0,1
Divorciado	13	0,6
**Tipo de vivienda**		
Solo	410	17,5
Con la familia	942	40,1
Pensión	58	2,5
República	794	33,8
Alojamiento estudiantil	145	6,2
**Ingreso familiar en salarios mínimos** ^‡^		
Hasta 3	1.380	58,7
4 o más	969	41,3
**Actividad laboral en el momento**	606	25,8
**Asistencia estudiantil en el momento**	621	26,4
**Beca estudiantil en el momento**	476	20,3
**Diagnóstico clínico de trastornos de salud mental**	1.337	56,9
**Uso de psicofármacos**	956	40,7
**Seguimiento con psiquiatra**		
No	1.638	69,7
Ya lo hacía antes de la pandemia	302	12,9
Comenzó durante/después de la pandemia	409	17,4
**Seguimiento con psicólogo**		
No	1.254	53,4
Ya lo hacía antes de la pandemia	461	19,6
Comenzó durante/después de la pandemia	634	27,0

*f = Frecuencia absoluta; ^†^% = Frecuencia relativa; ^‡^Valor del salario mínimo en el momento de la recolección de datos: R$1.320,00, 2023

De los 2.349 estudiantes que participaron en el estudio, el 76,8% (1.804) presentaban síntomas de ansiedad; el 77,3% (1.816) síntomas de estrés; el 77,2% (1.814) síntomas de depresión; y el 42,2% (994) TEPT, los cuales fueron medidos mediante las escalas empleadas.

El análisis bivariado demostró que todas las características definitorias de los diagnósticos de enfermería seleccionadas para su medición y reportadas por los estudiantes se asociaron de forma estadísticamente significativa con los síntomas de ansiedad, estrés, depresión y TEPT en la era post COVID-19 (Tabla 2).

La Tabla 3 presenta las características definitorias que se configuraron como predictoras de los trastornos de salud mental entre estudiantes universitarios de pregrado, a partir de los modelos de regresión logística multivariada.

**Tabla 2-:** Asociación entre características definitorias referidas por los estudiantes universitarios de pregrado y la presencia de síntomas de ansiedad, estrés, depresión y trastorno e estrés postraumático en la era post COVID-19 (n = 2.349). Viçosa, MG, Brasil, 2024

		Síntomas de ansiedad f* (% ^†^ )	Valor p	Síntomas de estrés f* (% ^†^ )	Valor p	Síntomas de depresión f* (% ^†^ )	Valor p	Trastorno de estrés postraumático f* (% ^†^ )	Valor p
**Características definitorias físicas**									
Alteración en el ciclo sueño-vigilia ^‡§^	No	329 (18,2)	**<0,001**	326 (18,0)	**<0,001**	335 (18,5)	**<0,001**	130 (13,1)	**<0,001**
	Sí	1475 (81,8)		1490 (82,0)		1479 (81,5)		864 (86,9)	
Sudoración aumentada ^‡^	No	900 (49,9)	**<0,001**	917 (50,5)	**<0,001**	940 (51,8)	**<0,001**	433 (43,6)	**<0,001**
	Sí	904 (50,1)		899 (49,5)		874 (48,2)		561 (56,4)	
Boca seca ^‡^	No	825 (45,7)	**<0,001**	871 (48,0)	**<0,001**	882 (48,6)	**<0,001**	391 (39,3)	**<0,001**
	Sí	979 (54,3)		945 (52,0)		932 (51,4)		603 (60,7)	
Patrón respiratorio alterado ^‡^	No	636 (35,3)	**<0,001**	657 (36,2)	**<0,001**	701 (38,6)	**<0,001**	284 (28,6)	**<0,001**
	Sí	1168 (64,7)		1159 (63,8)		1113 (61,4)		710 (71,4)	
Palpitaciones cardíacas ^‡||^	No	644 (35,7)	**<0,001**	694 (38,2)	**<0,001**	720 (39,7)	**<0,001**	308 (31,0)	**<0,001**
	Sí	1160 (64,3)		1122 (61,8)		1094 (60,3)		686 (69,0)	
Tensión muscular ^‡¶^	No	373 (20,8)	**<0,001**	362 (19,9)	**<0,001**	408 (22,5)	**<0,001**	168 (16,9)	**<0,001**
	Sí	1431 (79,3)		1454 (80,1)		1406 (77,5)		826 (83,1)	
Cefalea ^||^	No	641 (35,5)	**<0,001**	646 (35,6)	**<0,001**	6607 (36,8)	**<0,001**	296 (29,8)	**<0,001**
	Sí	1163 (64,5)		1170 (64,4)		1147 (63,2)		698 (70,2)	
Náuseas ^‡||^	No	1080 (59,9)	**<0,001**	1101 (60,6)	**<0,001**	1117 (61,6)	**<0,001**	545 (54,8)	**<0,001**
	Sí	724 (40,1)		715 (39,4)		697 (38,4)		449 (45,2)	
Opresión en el pecho ^‡^	No	591 (32,8)	**<0,001**	625 (34,4)	**<0,001**	646 (35,6)	**<0,001**	264 (26,6)	**<0,001**
	Sí	1213 (67,2)		1191 (65,6)		1168 (64,4)		730 (73,4)	
Diarrea ^‡||^	No	1235 (68,5)	**<0,001**	1245 (68,6)	**<0,001**	1260 (69,5)	**<0,001**	640 (64,4)	**<0,001**
	Sí	569 (31,5)		571 (31,4)		554 (30,5)		354 (994)	
Rubor facial ^‡^	No	1427 (79,1)	**<0,001**	1441 (79,4)	**<0,001**	1453 (80,1)	**<0,001**	756 (76,1)	**<0,001**
	Sí	373 (20,7)		371 (20,4)		357 (19,7)		238 (23,9)	
**Características definitorias comportamentales**									
Agitación psicomotora ^‡^	No	150 (8,3)	**<0,001**	131 (7,2)	**<0,001**	180 (9,9)	**<0,001**	61 (6,1)	**<0,001**
	Sí	1654 (91,7)		1685 (92,8)		1634 (90,1)		933 (93,9)	
Llanto fácil ^‡^	No	490 (27,2)	**<0,001**	483 (26,6)	**<0,001**	521 (28,7)	**<0,001**	214 (21,5)	**<0,001**
	Sí	1314 (72,8)		1333 (73,4)		1293 (71,3)		780 (78,5)	
Impaciencia aumentada ^¶^	No	219 (12,1)	**<0,001**	169 (9,3)	**<0,001**	214 (11,8)	**<0,001**	80 (8,0)	**<0,001**
	Sí	1585 (87,9)		1647 (90,7)		1600 (88,2)		914 (92,0)	
Productividad disminuida ^‡^	No	287 (15,9)	**<0,001**	292 (16,1)	**<0,001**	258 (14,2)	**<0,001**	123 (12,4)	**<0,001**
	Sí	1517 (84,1)		1524 (83,9)		1556 (85,8)		871 (87,6)	
Baja autoestima ^§^	No	266 (14,9)	**<0,001**	273 (15,1)	**<0,001**	246 (13,7)	**<0,001**	96 (9,7)	**<0,001**
	Sí	1525 (85,1)		1531 (84,9)		1556 (86,3)		898 (90,3)	
**Características definitorias cognitivas**									
Atención alterada ^‡||^	No	212 (11,8)	**<0,001**	201 (11,1)	**<0,001**	209 (11,5)	**<0,001**	87 (8,8)	**<0,001**
	Sí	1592 (88,2)		1615 (88,9)		1605 (88,5)		907 (91,2)	
Olvidos frecuentes ^‡^	No	291 (16,1)	**<0,001**	280 (15,4)	**<0,001**	296 (16,3)	**<0,001**	117 (11,8)	**<0,001**
	Sí	1513 (83,9)		1536 (84,6)		1518 (83,7)		877 (88,2)	
**Características definitorias emocionales**									
Preocupación excesiva ^‡§^	No	110 (6,1)	**<0,001**	96 (5,3)	**<0,001**	126 (6,9)	**<0,001**	24 (2,4)	**<0,001**
	Sí	1694 (93,9)		1720 (94,7)		1688 (93,1)		970 (97,6)	
Frustración ^§^	No	195 (10,8)	**<0,001**	190 (10,5)	**<0,001**	154 (8,5)	**<0,001**	68 (6,8)	**<0,001**
	Sí	1609 (89,2)		1626 (89,5)		1660 (91,5)		926 (93,2)	
Soledad ^§^	No	437 (24,2)	**<0,001**	421 (23,2)	**<0,001**	401 (22,1)	**<0,001**	178 (17,9)	**<0,001**
	Sí	1367 (75,8)		1395 (76,8)		1413 (77,9)		816 (82,1)	
Miedo ^‡§||^	No	305 (16,9)	**<0,001**	326 (18,0)	**<0,001**	325 (17,9)	**<0,001**	114 (11,5)	**<0,001**
	Sí	1499 (83,1)		1490 (82,0)		1489 (82,1)		880 (88,5)	
Rabia ^§||¶^	No	557 (30,9)	**<0,001**	521 (28,7)	**<0,001**	538 (29,7)	**<0,001**	225 (22,6)	**<0,001**
	Sí	1247 (69,1)		1295 (71,3)		1276 (70,3)		769 (77,4)	
Tristeza**	No	266 (14,7)	**<0,001**	273 (15,0)	**<0,001**	246 (13,6)	**<0,001**	68 (6,8)	**<0,001**
	Sí	1538 (85,3)		1543 (85,0)		1568 (86,4)		926 (93,2)	

*f = Frecuencia absoluta; ^†^% = Frecuencia relativa; características definitorias de los diagnósticos de enfermería; ^‡^Ansiedad; ^§^Síndrome del Estrés por Cambio; ^||^Síndrome Postraumático; ^¶^Sobrecarga de Estrés; ^**^Tristeza Crónica, según la taxonomía NANDA-I(8); valor p < 0,05 – estadísticamente significativo

**Tabla 3-:** Características definitorias que se configuran como predictoras de trastornos de salud mental en universitarios en la era post COVID-19 (n = 2.349). Viçosa, MG, Brasil, 2024

		Síntomas de ansiedad		Síntomas de estrés		Síntomas de depresión		Trastorno de estrés postraumático	
	Categorías	ORA* (IC 95% ^†^ )	Valor P	ORA* (IC 95% ^†^ )	Valor P	ORA* (IC 95% ^†^ )	Valor P	ORA* (IC 95% ^†^ )	Valor P
**Características definitorias físicas**									
Alteración en el ciclo sueño-vigilia ^§||^	No ^‡^	-	-	-	-	1		1	
	Sí	-	-	-	-	1,405 (1,054-1,872)	**0,021**	1,437 (1,111-1,858)	**0,006**
Sudoración aumentada ^§^	No ^‡^	1		1		-	**-**	1	
	Sí	1,463 (1,075-1,991)	**0,015**	1,382 (1,016-1,878)	**0,039**	-	**-**	1,355 (1,108-1,658)	**0,003**
Boca seca ^§^	No ^‡^	1		-	-	1		1	
	Sí	3,312 (2,369-4,630)	**<0,001**	-	-	1,613 (1,209-2,152)	**0,001**	1,551 (1,265-1,901)	**<0.001**
Patrón respiratorio alterado ^§^	No ^‡^	1		1		-	-	1	
	Sí	1,618 (1,170-2,238)	**0,004**	1,877 (1,353-2,604)	**<0,001**	-	-	1,373 (1,097-1,719)	**0,006**
Palpitaciones cardíacas ^§¶^	No ^‡^	1		1		-	-	1	
	Sí	3,316 (2,379-4,623)	**<0,001**	1,455 (1,058-2,002)	**0,021**	-	-	1,319 (1,062-1,638)	**0,012**
Tensión muscular ^§**^	No ^‡^	-	-	1		-	-	-	-
	Sí	-	-	1,444 (1,072-1,943)	**0,016**	-	-	-	-
Cefalea ^¶^	No ^‡^	-	-	1		-	-	1	
	Sí	-	-	1,298 (0,966-1,744)	0,083	-	-	1,299 (1,054-1,599)	**0,014**
Náuseas ^§¶^	No ^‡^	1		1		1		1	
	Sí	2,707 (1,822-4,020)	**<0,001**	1,628 (1,109-2,390)	**0,013**	1,952 (1,397-2,726)	**<0,001**	1,226 (0,933-1,514)	0,058
Opresión en el pecho ^§^	No ^‡^	1		-	-	-	-	-	-
	Sí	1,422 (1,041-1,941)	**0,027**	-	-	-	-	-	-
**Características definitorias comportamentales**									
Agitación psicomotora ^§^	No ^‡^	1		1		-	-	1	
	Sí	2,238 (1,614-3,104)	**<0,001**	3,233 (2,312-4,522)	**<0,001**	-	-	1,425 (1,009-2,013)	**0,045**
Llanto fácil ^§^	No ^‡^	1		1		-	-	1	
	Sí	1,550 (1,170-2,053)	**0,002**	1,813 (1,358-2,420)	**<0,001**	-	-	1,343 (1,076-1,678)	**0,009**
Impaciencia aumentada**	No ^‡^	-	-	1		1		-	-
	Sí	-	-	2,225 (1,601-3,093)	**<0,001**	1,319 0,953-1,825)	0,095	-	-
Productividad disminuida ^§^	No ^‡^	-	-	-	-	1		-	-
	Sí	-	-	-	-	2,449 (1,827-3,282)	**<0,001**	-	-
Baja autoestima ^||^	No ^‡^	1		1		1		1	
	Sí	2,026 (1,476-2,782)	**<0,001**	1,632 (1,189-2,239)	**0,002**	2,205 (1,649-2,949)	**<0,001**	1,696 (1,272-2,260)	**<0,001**
**Características definitorias cognitivas**									
Atención alterada ^§¶^	No ^‡^	-	-	1		-	-	-	-
	Sí	-	-	1,649 (1,169-2,325)	**0,004**	-	-	-	-
Olvidos frecuentes ^§^	No ^‡^	1		1		1		1	
	Sí	1,433 (1,061-1,934)	**0,019**	1,644 (1,197-2,257)	**0,002**	1,362 (1,009-1,838)	**0,043**	1,547 (1,189-2,013)	**0,001**
**Características definitorias emocionales**									
Preocupación excesiva ^§||^	No ^‡^	1		1		1		1	
	Sí	1,728 (1,209-2,471)	**0,003**	2,698 (1,868-3,897)	**<0,001**	1,539 (1,075-2,205)	**0,019**	2,825 (1,764-4,523)	**<0,001**
Frustración ^||^	No ^‡^	1		-	-	1		-	-
	Sí	1,412 (0,991-2,012)	0,056	-	-	2,454 (1,793-3,359)	**<0,001**	-	-
Soledad ^||^	No ^‡^	-	-	1		1		1	
	Sí	-	-	1,677 (1,237-2,273)	**0,001**	1,822 (1,378-2,407)	**<0,001**	1,420 (1,120-1,801)	**0,004**
Miedo ^§||¶^	No ^‡^	1		-	-	1		1	
	Sí	2,036 (1,508-2,748)	**<0,001**	-	-	1,305 (0,972-1,152)	**0,076**	1,677 (1,281-2,196)	**<0,001**
Rabia ^||¶**^	No ^‡^	-	-	1		1		1	
	Sí	-	-	2,213 (1,641-2,986)	**<0,001**	1,923 (1,446-2,557)	**<0,001**	1,597 (1,287-1,983)	**<0,001**
Tristeza ^††^	No ^‡^	1		1		1		1	
	Sí	1,487 (1,062-2,082)	**0,021**	1,572 (1,118-2,211)	**0,009**	2,557 (1,871-3,472)	**<0,001**	1,522 (1,091-2,123)	**0,013**

*ORA = *Odds Ratio* Ajustado; ^†^ IC 95% = Intervalo de confianza del 95%; ^‡^ Referencia para el ORA, características definitorias de los diagnósticos de enfermería; ^§^ Ansiedad; ^||^ Síndrome del Estrés por Cambio; ^¶^ Síndrome Postraumático; **Sobrecarga de Estrés; ^††^ Tristeza Crónica, según la taxonomía NANDA-I ^( 8 )^ ; valor p < 0.05 - estadísticamente significativo

## Discusión

Este estudio determinó características definitorias físicas, conductuales, cognitivas y emocionales de los diagnósticos de enfermería, según la taxonomía de la NANDA-I ^( 8 )^ , Ansiedad (00146); Sobrecarga de Estrés (00177); Síndrome Postraumático (00141); Síndrome del Estrés por Cambio (00114) y Tristeza Crónica (00137), que se configuraron como predictoras en aquellos estudiantes universitarios con trastornos de salud mental en la era post COVID-19. Tanto las características definitorias de los diagnósticos de enfermería observados como los trastornos de salud mental fueron altamente prevalentes en esta población, lo que hace que estos hallazgos sean preocupantes. 

Los problemas de salud mental se agravaron significativamente durante la pandemia de COVID-19 y continuaron después de su término, incluso entre estudiantes de pregrado. Investigaciones realizadas con este público ya han reportado la prevalencia de estos problemas a nivel mundial en la era post COVID-19 ^( 6 - 7 , 9 )^ . Sin embargo, encontramos mayores prevalencias de estos trastornos de salud mental en los estudiantes brasileños. Esto indica el gran sufrimiento mental vivido en la era post pandemia y la urgente necesidad de establecer políticas de atención que prioricen su salud mental. 

Con respecto a las características definitorias físicas, la sudoración aumentada, el patrón respiratorio alterado y las palpitaciones cardíacas emergieron como predictoras de ansiedad, estrés y TEPT; la boca seca, de ansiedad, TEPT y depresión; las náuseas, de ansiedad, estrés y depresión; la opresión en el pecho, de ansiedad y depresión; la alteración en el ciclo sueño-vigilia, de TEPT y depresión; la tensión muscular, de estrés; y la cefalea, de TEPT.

Según la NANDA-I ^( 8 )^ , todos estos signos y síntomas físicos, con excepción de la cefalea, son características definitorias del diagnóstico Ansiedad. El diagnóstico Síndrome del Estrés por Cambio también presenta como característica definitoria la referencia a síntomas físicos intensificados. El diagnóstico Sobrecarga de Estrés incluye la expresión de tensión. Las palpitaciones cardíacas y la cefalea se incluyen en el diagnóstico Síndrome Postraumático. El diagnóstico Tristeza Crónica no presenta características definitorias físicas. Estos hallazgos reafirman que estas características definitorias están efectivamente presentes en personas con ansiedad, estrés y depresión y, además, pueden conducir al desarrollo de estas condiciones clínicas, ya que se configuraron como variables predictoras en el presente estudio. 

En el contexto de la pandemia de COVID-19, las alteraciones en el patrón de sueño fueron frecuentes entre los estudiantes universitarios ^( 17 )^ . En el presente estudio, esta prevalencia también fue expresiva para los cuatro trastornos investigados (superior al 80,0%), lo que demuestra que las alteraciones en el ciclo sueño-vigilia se mantuvieron en la era post COVID-19. Estos datos son preocupantes, ya que la alteración del patrón de sueño tiene una correlación directa con los problemas de salud mental en universitarios ^( 12 )^ . 

Manifestaciones como sudoración aumentada, boca seca, parámetros cardíacos y respiratorios alterados, y alteraciones gastrointestinales como las náuseas, también pueden estar presentes en personas con ansiedad ^( 8 )^ . Estas manifestaciones también son evaluadas en instrumentos validados como la DASS-21 ^( 14 )^ para medir ansiedad, estrés y depresión, y en la IES-R para la evaluación del TEPT ^( 15 )^ . Esto reafirma la importancia de valorar dichas características. 

En el presente estudio, las prevalencias de boca seca, palpitaciones cardíacas y patrón respiratorio alterado fueron elevadas entre los estudiantes que presentaron ansiedad (54,3%, 64,3% y 64,7%, respectivamente). Y, particularmente, los parámetros cardíacos y respiratorios también fueron relevantes entre quienes presentaron TEPT (69% y 71,4%, respectivamente) y estrés (61,8% y 63,8%, respectivamente). La activación del eje hipotálamo-hipófisis-adrenal y del sistema nervioso simpático en situaciones de estrés justifica estas manifestaciones ^( 18 )^ . En el contexto post pandémico, las elevadas prevalencias de TEPT y estrés en los estudiantes revelan la importancia de evaluar tales manifestaciones en este público. 

La tensión muscular emergió como característica definitoria predictora de la presencia de estrés, con una prevalencia significativa en los estudiantes del presente estudio (80,1%). Como el sistema nervioso autónomo inerva prácticamente todos los sistemas orgánicos del cuerpo, la activación de la respuesta simpática puede provocar diversas alteraciones fisiológicas, como, por ejemplo, el aumento de la tensión y del dolor muscular ^( 19 )^ . En estudiantes universitarios, durante la pandemia, investigadores observaron una alta prevalencia de tensión en los músculos faciales, y los niveles de estrés fueron significativamente más altos ^( 20 )^ . De la misma manera, la cefalea también puede presentarse y estar asociada a elevados niveles de estrés en estudiantes ^( 21 )^ . De hecho, en el presente estudio, esta característica definitoria fue predictora del desarrollo de TEPT, con una alta prevalencia entre los estudiantes (70,2%). 

Otro síntoma físico que se configuró como característica definitoria predictora de ansiedad y depresión fue la sensación de opresión en el pecho, con una prevalencia del 67,2% en estudiantes con ansiedad y del 64,4% en estudiantes con depresión. Un estudio realizado con estudiantes universitarios encontró una asociación significativa entre la presencia de ansiedad y el síntoma de opresión en el pecho o en el corazón; lo mismo no se observó para la depresión ^( 22 )^ . Es importante destacar que este síntoma aparece como característica definitoria del diagnóstico Ansiedad en la NANDA-I ^( 8 )^ . Sin embargo, debido a su alta prevalencia en estudiantes con síntomas de depresión y al hecho de haber emergido como variable predictora para esta condición, se sugiere evaluar la posibilidad de incluir este síntoma como característica definitoria del diagnóstico Tristeza Crónica. 

Al considerar que las características definitorias físicas son más fácilmente perceptibles, pueden actuar como señales de alerta para estas condiciones. En el contexto universitario, por ejemplo, cuando los docentes identifiquen la presencia de estas manifestaciones en los estudiantes dentro del aula, se debe prestar mayor atención a su salud mental, de modo que los problemas emocionales puedan ser identificados en etapas iniciales y se implementen intervenciones tempranas.

Entre las características definitorias conductuales identificadas en los estudiantes, la baja autoestima fue predictora de ansiedad, estrés, depresión y TEPT; la agitación psicomotora y el llanto fácil fueron predictoras de ansiedad, estrés y TEPT; la impaciencia aumentada lo fue para el estrés; y la productividad disminuida, para la depresión. Según la NANDA-I ^( 8 )^ , la baja autoestima es una característica definitoria del diagnóstico Síndrome del Estrés por Cambio; la agitación psicomotora, el llanto fácil y la productividad disminuida son características del diagnóstico Ansiedad; y la impaciencia aumentada se configura como característica definitoria de Sobrecarga de Estrés. 

La baja autoestima puede entenderse como una percepción negativa del propio valor, la autoaceptación, el autorrespeto, la competencia y la actitud hacia uno mismo ^( 8 )^ . En el contexto universitario, la baja autoestima es un factor que influye en niveles más altos de depresión ^( 23 )^ , además de comprometer significativamente el rendimiento académico ^( 24 )^ . Esta situación se agravó con la pandemia y persiste en el contexto post pandémico, como se observa en la prevalencia expresiva de baja autoestima, que osciló entre el 84,9% y el 90,1% entre los estudiantes con trastornos de salud mental en este estudio. Por ello, la evaluación de la autoestima en los estudiantes puede contribuir al establecimiento más preciso de diagnósticos relacionados con la salud mental y, en consecuencia, a la implementación de intervenciones más eficaces. 

La agitación psicomotora y el llanto fácil también fueron altamente prevalentes (aproximadamente 92,0% y 75,0%, respectivamente) entre los estudiantes con ansiedad, estrés y TEPT. La DASS-21 ^( 14 )^ incluye, en el constructo de ansiedad, la presencia de temblores, por ejemplo, en las manos, y en el constructo de estrés, dificultades para calmarse y relajarse, así como agitación. Estos síntomas están relacionados con la agitación psicomotora, lo que refuerza la importancia de su evaluación en personas con síntomas de ansiedad, estrés y en situaciones postraumáticas. Por otro lado, el llanto fácil, que emergió como predictor de estas condiciones, también está presente en la DASS-21 ^( 14 )^ , aunque de forma indirecta, como “sentí que estaba un poco emotivo/demasiado sensible” en el constructo depresión. Se reafirma la necesidad de detectar estas características definitorias en los estudiantes, quienes se vieron fuertemente afectados a nivel psicológico por la pandemia de COVID-19. 

La impaciencia aumentada también fue expresiva entre los estudiantes que presentaron síntomas de estrés (90,7%) en el contexto post pandémico. Este también es un síntoma evaluado por la IES-R en personas con TEPT (“me sentía irritable y enojado”) ^( 15 )^ . Las diversas consecuencias de la pandemia en la modalidad de enseñanza llevaron a los estudiantes a desarrollar sentimientos de impaciencia, molestia y hostilidad ^( 25 )^ , lo cual puede predisponer al desarrollo de estrés, como se evidenció en el presente estudio. Ante esto, resulta importante trabajar estrategias de afrontamiento y alivio del estrés con esta población, de forma que esos sentimientos negativos no afecten el desempeño académico del estudiante. 

Otro síntoma frecuente entre los estudiantes de este estudio, que se configuró como predictor de depresión, fue la productividad disminuida (85,8%). Se destaca que la falta de voluntad para realizar actividades cotidianas es una característica común de la depresión. En el contexto universitario, esta falta de voluntad puede reflejarse en una disminución de la productividad, con un consecuente impacto en el rendimiento académico. Sin embargo, en la NANDA-I, el diagnóstico Tristeza Crónica no presenta esta característica definitoria ^( 8 )^ . Debido a la alta prevalencia evidenciada en este estudio y su relación directa con la depresión, se sugiere su posible inclusión. 

Entre los signos y síntomas cognitivos, el olvido frecuente fue predictor de ansiedad, estrés, TEPT y depresión; y la atención alterada, de estrés y TEPT. Según la NANDA-I, el olvido se configura como característica definitoria del diagnóstico Ansiedad; y la atención alterada, como característica de Ansiedad y del diagnóstico Síndrome Postraumático. Además, la IES-R incluye los problemas de concentración como parte del cuadro del TEPT ^( 15 )^ , lo que refuerza la necesidad de evaluar estos síntomas. 

Durante la pandemia de COVID-19, la dificultad de concentración fue frecuente entre estudiantes universitarios, con una prevalencia cercana al 89% ^( 17 )^ . Los problemas cognitivos persistieron hasta la era post pandémica, hecho comprobado en el presente estudio, que encontró una prevalencia de olvido frecuente superior al 80% en todos los constructos evaluados, y de atención alterada del 88,9% y 91,2% en estudiantes con estrés y TEPT, respectivamente. Estos hallazgos son importantes, ya que la presencia de estas características definitorias puede comprometer el rendimiento académico de los estudiantes, reducir su motivación para el estudio y, en última instancia, contribuir a la deserción escolar. 

Entre las características definitorias emocionales, la preocupación excesiva y la tristeza fueron predictoras de ansiedad, estrés, TEPT y depresión; la soledad y la rabia, de estrés, TEPT y depresión; el miedo, de ansiedad y TEPT; y la frustración, de depresión.

En el presente estudio, se observó una alta prevalencia de sentimientos de preocupación (>93,0% en todos los constructos) y de tristeza (≥85,0% en todos los constructos) entre los estudiantes. Un estudio realizado con 195 estudiantes universitarios, que buscó comprender los efectos de la pandemia en su salud mental, encontró que la preocupación por el rendimiento académico y los pensamientos excesivos y depresivos también estaban presentes en esta población; además, estos pensamientos contribuyeron a la aparición de ideas suicidas ^( 17 )^ . Ante tales hallazgos, queda claro que estos sentimientos aún persisten en el contexto post pandémico y que, cuando se intensifican, pueden comprometer la salud mental de los estudiantes y llevarlos a comportamientos de riesgo. Por lo tanto, al evaluar la salud mental de los estudiantes, se sugiere observar la presencia de estos sentimientos al considerar todos los diagnósticos de enfermería estudiados aquí. 

Los sentimientos de soledad ^( 17 )^ y de rabia ^( 26 )^ , presentes entre estudiantes durante la pandemia, también mostraron una alta prevalencia en la era post COVID-19. De hecho, más del 75,0% y del 69,0% de los estudiantes de este estudio presentaron estos síntomas, respectivamente. Según la NANDA-I, estos sentimientos están presentes en los diagnósticos de Síndrome del Estrés por Cambio, Síndrome Postraumático y Sobrecarga de Estrés ^( 8 )^ . Sin embargo, en el presente estudio también emergieron como predictores de depresión en estudiantes de pregrado, por lo que se sugiere su evaluación también en casos de tristeza. 

El miedo, también reportado por estudiantes universitarios en el contexto de la pandemia ^( 17 )^ , se manifestó de forma muy frecuente en la era post COVID-19, como lo demuestra nuestro estudio, especialmente entre los estudiantes con ansiedad (83,1%) y con TEPT (88,5%). El miedo puede influir en múltiples aspectos de la vida y la salud de las personas ^( 27 )^ . Durante la pandemia, esta reacción desencadenó serias consecuencias fisiopatológicas, sociales, conductuales y mentales ^( 27 )^ . De hecho, un estudio señaló que la mayor prevalencia del miedo a la COVID-19 se relacionó con peor calidad del sueño, peor percepción de salud, tristeza, mayor estrés, síntomas depresivos e ideación suicida ^( 27 )^ . Por lo tanto, comprender y mitigar el miedo en la población universitaria es una gran preocupación y un punto clave para las intervenciones ^( 28 )^ . Cabe destacar que, según la NANDA-I ^( 8 )^ , este sentimiento está presente en los diagnósticos Ansiedad, Síndrome del Estrés por Cambio y Síndrome Postraumático, lo que demuestra que los resultados encontrados están alineados con la taxonomía. 

Por último, la frustración fue otro síntoma expresivo en el presente estudio, con una prevalencia del 91,5% en estudiantes con depresión. Durante la pandemia, los estudiantes expresaron frustraciones relacionadas con las clases remotas, el acceso a tecnologías, la interrupción de actividades de investigación y extensión, así como en aspectos familiares, sociales, emocionales, conductuales y financieros de la vida ^( 29 )^ . Con los hallazgos aquí presentados, se percibe que este sentimiento persiste en la era post COVID-19. Garantizar, por tanto, que las frustraciones de los estudiantes y las barreras al éxito académico sean reconocidas y tenidas en cuenta puede ayudar a prevenir el abandono de la educación superior ^( 29 )^ . 

A la luz de los resultados de esta investigación, se sugiere que las características definitorias físicas, conductuales, cognitivas y emocionales que emergieron como predictoras de ansiedad, estrés, TEPT y depresión en estudiantes de pregrado en la era post COVID-19 sean estudiadas más profundamente como posibles características definitorias de los respectivos diagnósticos Ansiedad, Síndrome del Estrés por Cambio, Síndrome Postraumático, Sobrecarga de Estrés y Tristeza Crónica. Estas características pueden proporcionar más indicios diagnósticos y, en consecuencia, facilitar la implementación de intervenciones efectivas para este grupo poblacional.

Este estudio presenta limitaciones que deben ser consideradas. En primer lugar, la recolección de datos en formato en línea impidió el cálculo preciso de la tasa de respuesta; además, los estudiantes sin acceso a internet no pudieron participar. En futuras investigaciones, la recolección presencial de datos podría evitar este sesgo de selección. En segundo lugar, se utilizaron cuestionarios de autoinforme para evaluar los síntomas relacionados con los trastornos de salud mental, así como la presencia de características definitorias de los diagnósticos de enfermería estudiados. Para estudios futuros, se sugiere la realización de diagnósticos clínicos por parte de médicos y la evaluación de las características definitorias por enfermeros, de modo que estos datos no se basen únicamente en la autoevaluación de los participantes. En tercer lugar, este estudio se limitó a una sola región del país, lo que puede restringir en cierta medida la generalización de los resultados a otras regiones o países. Sin embargo, el tamaño muestral expresivo, proveniente de 15 *campi* (en diferentes ciudades) y 94 cursos de pregrado, aumentó la confiabilidad y validez de las comparaciones realizadas. Además, las asociaciones con ORA inferior a 2,0 deben ser interpretadas con cautela, ya que pueden generar asociaciones falso positivas ^( 30 )^ . También se sugiere considerar en los análisis estadísticos el semestre académico, ya que los estudiantes de diferentes periodos pueden presentar características distintas. Por último, se destaca que no se utilizó la versión más reciente de la NANDA-I, pues en el momento de la recolección de datos aún no había sido publicada. No obstante, los investigadores observaron que las características definitorias investigadas no estaban contempladas en dicha versión. 

A pesar de las limitaciones, este estudio aporta avances al conocimiento científico, pues se encuentra entre los primeros en identificar la prevalencia de trastornos relacionados con la salud mental en estudiantes de pregrado en la era post COVID-19 en Brasil. En particular, es relevante para el área de la enfermería, ya que evidenció características definitorias de diagnósticos de enfermería que se configuraron como predictoras de ansiedad, estrés, depresión y TEPT. Estos hallazgos pueden ayudar a los enfermeros a perfeccionar la evaluación de trastornos de salud mental en la población universitaria, así como a implementar intervenciones eficaces para la promoción de la salud mental.

Además, cabe resaltar que la versión más reciente de la NANDA-I, lanzada en abril de 2024 ^( 31 )^ , no contempla los diagnósticos de Síndrome del Estrés por Cambio, Sobrecarga de Estrés y Tristeza Crónica. Al considerar la alta prevalencia de las características definitorias de estos diagnósticos en una muestra expresiva de estudiantes de pregrado, y que estas fueron predictoras de ansiedad, estrés, depresión y TEPT, este estudio valida su ocurrencia. Por lo tanto, se sugiere la reincorporación de dichos diagnósticos, con sus respectivas características definitorias, en la taxonomía de la NANDA-I. 

## Conclusión

Este estudio evidenció características definitorias físicas, conductuales, cognitivas y emocionales presentes en los diagnósticos de enfermería Ansiedad, Síndrome del Estrés por Cambio, Síndrome Postraumático, Sobrecarga de Estrés y Tristeza Crónica, según la NANDA-I, que se configuraron como predictoras del desarrollo de trastornos de salud mental en estudiantes universitarios en la era post COVID-19, tales como: alteración en el ciclo sueño-vigilia, sudoración aumentada, boca seca, patrón respiratorio alterado, palpitaciones cardíacas, tensión muscular, cefalea, náuseas, opresión en el pecho, agitación psicomotora, llanto fácil, impaciencia aumentada, productividad disminuida, baja autoestima, atención alterada, olvidos frecuentes, preocupación excesiva, frustración, soledad, miedo, rabia y tristeza.

## Data Availability

El conjunto de datos de este artículo está disponible en la página de la RLAE en el repositorio SciELO Data, en el enlace https://doi.org/10.48331/scielodata.D4GINI
